# Pharmacokinetic Profiles of Active Ingredients and Its Metabolites Derived from Rikkunshito, a Ghrelin Enhancer, in Healthy Japanese Volunteers: A Cross-Over, Randomized Study

**DOI:** 10.1371/journal.pone.0133159

**Published:** 2015-07-17

**Authors:** Hiroyuki Kitagawa, Masaya Munekage, Takashi Matsumoto, Chiharu Sadakane, Miwako Fukutake, Katsuyuki Aoki, Junko Watanabe, Kazuya Maemura, Tomohisa Hattori, Yosio Kase, Yasuhito Uezono, Akio Inui, Kazuhiro Hanazaki

**Affiliations:** 1 Department of Surgery, Kochi Medical School, Kochi University, Kochi, Japan; 2 Tsumura Research Laboratories, Kampo Scientific Strategies Division, Tsumura & Co., Ibaraki, Japan; 3 Kampo Formulations Development Center, Production Division, Tsumura & Co., Ibaraki, Japan; 4 Division of Cancer Pathophysiology, National Cancer Center Research Institute, Tokyo, Japan; 5 Department of Psychosomatic Internal Medicine, Kagoshima University Graduate School of Medical and Dental Sciences, Kagoshima, Japan; Monash University, AUSTRALIA

## Abstract

**Background:**

Rikkunshito, a traditional Japanese (Kampo) medicine, has been used to treat upper gastrointestinal disorders such as functional dyspepsia and gastroesophageal reflux. This study investigated the exposure and pharmacokinetics of the ingredients of rikkunshito in healthy volunteers.

**Methods and Results:**

First, an exploratory nonrandomized, open-label, one-period, noncrossover study using four healthy Japanese volunteers to detect 32 typical ingredients of rikkunshito in plasma and urine. As a result, 18 or 21 of 32 ingredients was detected in plasma or urine samples after oral administration of rikkunshito (7.5 g/day). Furthermore, a randomized, open-label, three-arm, three-period, crossover study using 21 subjects was conducted to determine the amounts of exposure and pharmacokinetic parameters of nine ingredients derived from rikkunshito (atractylodin, atractylodin carboxylic acid, pachymic acid, 3,3′,4′,5,6,7,8-heptamethoxyflavone, naringenin, nobiletin, liquiritigenin, isoliquiritigenin, and 18β-glycyrrhetinic acid) after oral administration of rikkunshito at three different doses (2.5, 5.0, or 7.5 g/day) during each period. The pharmacokinetic profiles of the nine ingredients in plasma were characterized. The geometric means (95% confidence interval) for the Cmax of the ingredients at a dose of 7.5 g were 1570 (1210–2040), 14,300 (12,200–16,800), 91.0 (71.8–115), 105 (75.6–144), 1150 (802–1650), 35.9 (24.6–52.5), 800 (672–952), 42.8 (30.4–60.3), and 55,600 (39,600–78,100) pg/mL, respectively, and for the AUC_0–last_ were 1760 (1290–2390), 12700 (11,100–14,600), 1210 (882–1650), 225 (157–322), 4630 (2930–7320), 35.7 (20.4–62.7), 4040 (3260–5010), 122 (88.2–168), and 832,000 (628,000–1,100,000) pg·h/mL respectively.

**Conclusions:**

We identified the ingredients of rikkunshito that are absorbed in humans. Furthermore, we determined the pharmacokinetics of nine ingredients derived from rikkunshito. This information will be useful for elucidating the pharmacological effects of rikkunshito.

**Trial Registration:**

Japan Pharmaceutical Information Center #CTI-121801 and -142522

## Introduction

Rikkunshito is a traditional Japanese medicine (called Kampo) in Japan. It is approved for medical use by the Japanese Ministry of Health and Welfare and has been reported to show efficacy for treating upper gastrointestinal disorders, such as functional dyspepsia [1, 2] and gastroesophageal reflux [3, 4], in multicenter double blind trials. A Kampo formulation is a combined extract of crude drugs and contains various components. For this reason, it has been generally recognized as complex for the mechanism of action to be linked to any particular active ingredient in a Kampo formulation. Mechanistic and pharmacokinetic studies of Kampo drugs have mainly progressed on the basis of recent basic studies [5–8]. Rikkunshito is an oral formulation that has already been used widely in clinical settings; therefore, it is important to understand the pharmacokinetics of its active ingredients.

Ghrelin is the sole peripheral hormone [9] that triggers appetite increase. Other functions of ghrelin, such as promoting growth hormone secretion and digestive tract functions, have also been found [10–12]. Ghrelin, which is produced mainly by X/A-like cells that reside in the gastric mucous membrane, binds to the ghrelin receptor (GHS-R) localized in vagus nerve endings and transmits a feed-promoting signal to the central nerves. A small amount of ghrelin is also produced in the central nervous system and other regions [9, 13]. In recent years, it has been discovered that stimulation of serotonin receptors, the 5-hydroxytryptamine (5-HT) 2B receptor (5-HT_2B_R) and 2C receptor (5-HT_2C_R), plays an important role in regulating ghrelin secretion in the stomach and hypothalamus [14, 15]. Rikkunshito and its ingredients promote ghrelin secretion by their antagonistic actions toward 5-HT_2B_R and 5-HT_2C_R [14, 16]. Owing to this research, GHS-R signal potentiation [1] or suppression of ghrelin metabolism [17] by the action of rikkunshito and its ingredients has become clear (Table 1). Understanding of the postadministration pharmacokinetics of active ingredients is critical to evaluate the efficacy and safety of rikkunshito. However, no pharmacokinetic study has determined whether active ingredients of rikkunshito, known as ghrelin enhancers, can be absorbed and reach the site of pharmacological action. Therefore, the contribution of these ingredients to the efficacy of rikkunshito is not completely understood.

**Table 1:** Major ingredients and their biological activities associated with ghrelin enhancer activity in rikkunshito.

Source	Major active ingredients	Known pharmacology activities
*Citri unshiu pericarpium*	Hesperetin	Ghrelin secretion promoting activity [14, 15]
	Heptamethoxyflavone	
*Glycyrrhizae radix*	Isoliquiritigenin	
*Atractylodis lanceae rhizoma*	Atractylodin	Ghrelin signal enhancement effect [1]
*Poria*	Pachymic acid	Ghrelin metabolizing enzyme inhibitory effect [17]

Although some previous reports are available on blood pharmacokinetics of the *Citri unshiu pericarpium*-derived ingredient naringenin and *Glycyrrhizae radix*-derived GA, blood orange juice [18], diammonium glycyrrhizinate capsules, or aforementioned compounds as single agents were administered in these studies [19, 20], and thus, the blood kinetics of individual ingredients after the administration of rikkunshito remains unknown. A kampo formulation comprises multiple crude drugs, and absorption and metabolism of ingredients may vary depending on the combination of the constituent crude drugs. Therefore, it is important to understand the pharmacokinetics of individual ingredients when administered as rikkunshito. In this study, we conducted a pharmacokinetic study of rikkunshito in humans with a particular focus on ingredients involved in the ghrelin enhancer effect. We first conducted an exploratory pharmacokinetic study of four healthy adult volunteers to identify the typical 32 ingredients (S1 Table) detected in the plasma or urine. Next, a randomized crossover study was conducted to investigate the pharmacokinetics of eight active ingredients derived from rikkunshito, which were selected with reference to the exploratory pharmacokinetic study and its pharmacological effect, in the plasma after a single oral administration of a clinical dose of rikkunshito in 21 healthy adult volunteers. We also measured atractylodin carboxylic acid, an atractylodin metabolite pharmacologically as potent as atractylodin (S1 Fig), although it was not measured in the exploratory pharmacokinetic study. Furthermore, the pharmacokinetic parameters of each ingredient were calculated based on the results.

## Methods

### Chemicals and reagents

Tsumura rikkunshito extract granules for prescription (product code TJ-43, Tsumura & Co. lot numbers E24652 and H05142, Tokyo, Japan) were used for the investigational product. It was manufactured according to GMP, and adapted to factory release test. The sample of the investigational drug used in this study is retained in Tsumura & Co. 7.5 g of this herbal preparation contains 4.0 g of dried extract obtained by spray drying of a hot water extract of a mixture of eight crude drugs: 4.0 g of *Atractylodis lanceae rhizoma* (Compositae; atractylodes lancea rhizome), 4.0 g of *Ginseng radix* (Araliaceae; ginseng), 4.0 g of *Pinelliae tuber* (Araceae; pinellia tuber), 4.0 g of *Poria* (Polyporaceae; poria sclerotium), 2.0 g of *Zizyphi fructus* (Rhamnaceae; jujube), 2.0 g of *C*. *unshiu pericarpium* (Rutaceae; citrus unshiu peel), 1.0 g of *G*. *radix* (Leguminosae; glycyrrhiza), and 0.5 g of *Zingiberis rhizoma* (Zingiberaceae; ginger). The standard components contained in rikkunshito and digoxin were supplied by Tsumura & Co. Atractylodin and atractylenolide III were supplied by Tsumura & Co. and Wako Pure Chemical Industries, Ltd. (Osaka, Japan). Erythromycin was purchased from Wako Pure Chemical Industries, Ltd. (±)-Warfarin-d5 was purchased from C/D/N ISOTOPES INC. (Pointe-Claire, Quebec, Canada). Other chemicals were purchased from commercial sources.

### Ethics statement

The trials were conducted at the Kochi Medical School in two periods: first trial, between April 2012 and March 2013 and the second trial, between September 2013 and May 2014, and these were approved by the Ethical Committee Kochi Medical School. The trials were registered at the Japan Pharmaceutical Information Center (JAPIC; #CTI-121801 and -142522). The trials were conducted in accordance with ethical norms prescribed in the Declaration of Helsinki and good clinical practice guidelines. All subjects read and signed the informed consent form prior to entering the study.

### Clinical trial design

**Fig 1:**
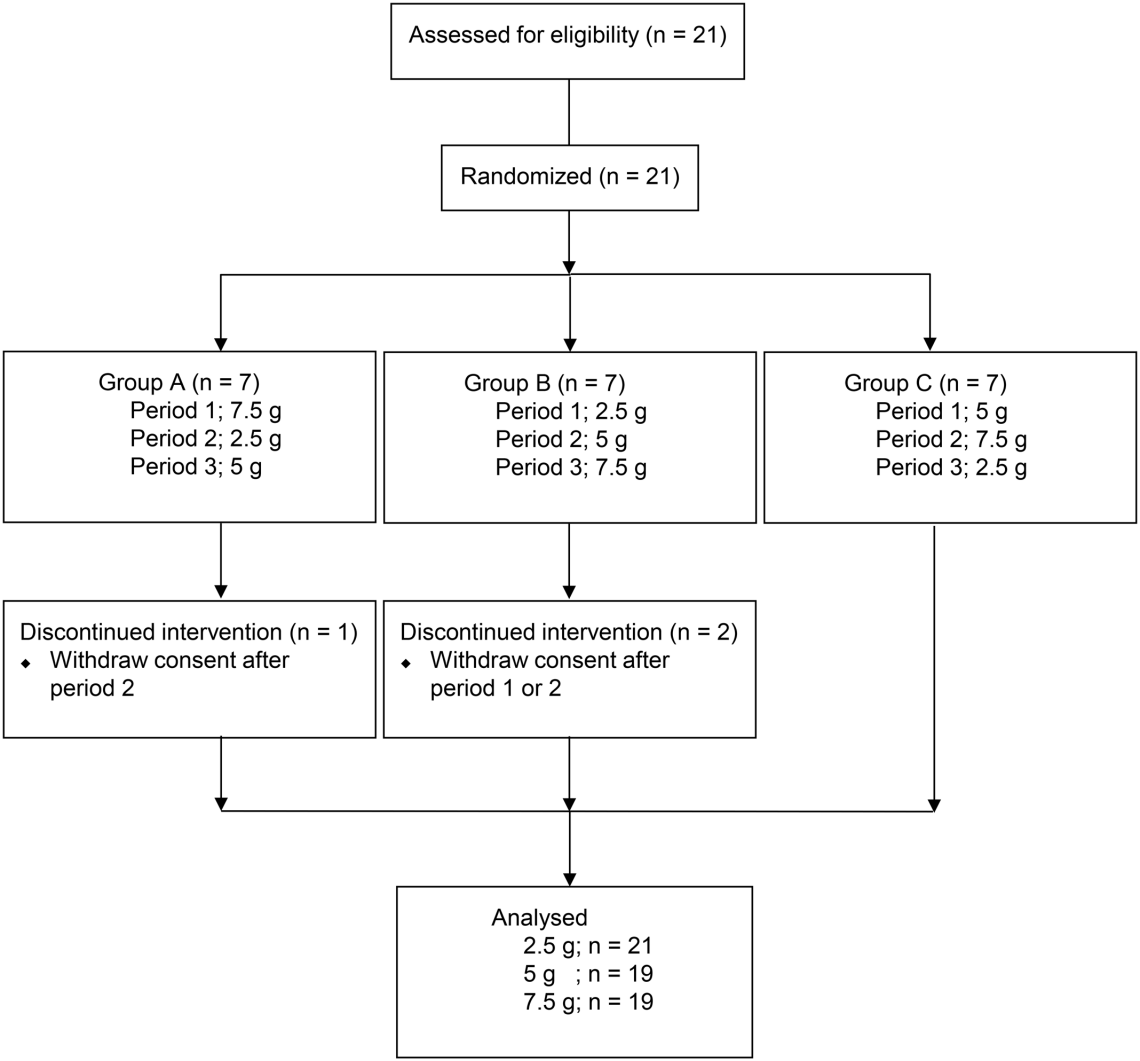
Flow diagram of participants through the randomized study

The first trial was a nonrandomized, open-label, one-period design. The second trial was a randomized, open-label, three-arm, three-period design (Fig 1). With respect to inclusion criteria, the studies included healthy Japanese adults between 20 and 45 years of age with a body mass index between 18 and 25 who were willing and able to comply with the study requirements. Exclusion criteria for this study included a history of significant liver, heart, or vessel disease and consumption of supplements that contained any rikkunshito ingredients or any drug within 3 days to 1 week before the first dose. Other standard exclusion criteria included relevant allergies, pregnant or nursing females, and any alcohol or nicotine use. The sample size was set as more than 15 based on the Ministry of Health, Labour and Welfare’s guidelines. Subjects allocated to a screening test were randomized to one of three groups by the central allocation system (allocation ratio 1:1:1). A random sequence was generated using a computer. The safety endpoint was evaluated in all volunteers. This endpoint was defined on the basis of a physician’s judgement after examination or observation that a serious adverse event had occurred. Adverse events included death, a life-threatening event, an event requiring hospitalization for treatment or an extended stay in hospital, an event resulting in permanent or temporary disability or dysfunction, an event resulting in a congenital abnormality, or any other serious medical phenomenon. With respect to side effects, no specific conditions were defined, and all adverse events for which no causal relationship with the drug could be excluded were deemed to be side effects. In each stage during the 48-h period following rikkunshito administration, the physician monitored the patient for subjective symptoms, objective findings, swelling, body temperature (axillary), blood pressure (sitting), and pulse (sitting). The physician also conducted a hematological examination to measure the patient’s red blood cell, leucocyte and platelet counts, hemoglobin level, and hematocrit value. A biochemical examination was conducted to determine total protein, blood urea nitrogen, creatinine, uric acid, aspartate aminotransferase, alanine aminotransferase, total bilirubin, alkaline phosphatase, γ-glutamyltranspeptidase albumin, prothrombin time, total cholesterol, C-reactive protein, and potassium to identify any abnormalities in comparison with the subject’s condition prior to administration. All participants fasted for 12 h before administration and 4 h after administration of the study drug. The subjects ate a controlled diet that did not contain rikkunshito ingredients from 3 days before the start of the study till the day of completion.

In the first exploratory trial, 20-mL blood samples were collected from the cutaneous vein in the subjects’ forearm via a blood sampling tube that contained heparin at 0 (preadministration), 0.5, 1, 2, 4, and 8 h after a single oral administration of rikkunshito (Lot E24652). The dose of rikkunshito used in this trial was 7.5 g/day, which is the clinical daily dose, to widely explore rikkunshito-derived ingredients that enter the blood. Along with plasma, urine samples were collected at three points: between dinner and bedtime (preadministration), between 0 and 4 h after administration, and between 4 and 8 h after administration. The blood was immediately centrifuged at 1700 ×*g* for 10 min to obtain plasma. Plasma and urine fractions were stored at −80°C or lower until analysis.

In the second quantitative trial, 12-mL blood samples were collected at 0 (preadministration), 0.25, 0.5, 1, 2, 3, 4, 6, 8, 10, 12, 24, and 48 h after a single oral administration of rikkunshito (Lot H05142). A total of 3 daily doses were used in this trial, i.e., the clinical dose per administration (2.5 g) as the lowest dose, the clinical daily dose (7.5 g) as the highest dose, and an intermediate dose (5.0 g), to broadly discuss the pharmacokinetic characteristics of the ingredients measured. The trial participants were hospitalized overnight on the day they ingested rikkunshito, and returned home the following day after blood samples were collected at the 24-h time point. The participants returned to the hospital to provide blood samples at 48 h. The blood was immediately centrifuged at 1700 ×*g* for 15 min to obtain plasma. Plasma fractions were stored at −20°C or lower until analysis.

### Analysis of rikkunshito ingredients

Plasma, urine, and rikkunshito formulation samples were analyzed for rikkunshito ingredients concentrations using a liquid chromatography–mass spectrometry with tandem mass spectrometer assay (LC–MS/MS; API5000, Triple Quad 6500, or QTRAP5500; AB Sciex, Tokyo, Japan) or gas chromatography–mass spectrometry. Further information of analytical methods can be found at S1 Appendix.

### Method validation

Validation of the procedure for analysis active ingredients was conducted with human plasma to evaluate the method with respect to specificity, recovery, intra- and inter-day reproducibility, calibration curve, stability in blood, short- and long-term stability, post-preparative stability, freeze–thaw stability, dilution integrity, matrix effect, carryover, limit of quantification, and stability in the standard solution.

### Pharmacokinetics analysis

Plasma pharmacokinetic data were analyzed by noncompartmental modeling using Phoenix WinNonlin (version 6.3, Certara L.P., St. Louis, MO) to determine various pharmacokinetic constants including the maximum concentration (*C*_max_), time to maximum concentration (*t*_max_), apparent elimination half-life (*t*_1/2_), and area under the plasma concentration-time curve from zero to last observation time (AUC_*0–last*_). The *t*_1/2_ was divided by log_e_2/*k*e, where *k*e is the terminal elimination (at least three data points on the descending linear limb) rate constant. The plasma concentration, *C*_max_, AUC_0–last_, and *t*_1/2_ of the target components in each group are presented as the geometric mean [95% confidence interval (CI)]. The *t*_max_ data are presented as medians with range from minimum to maximum.

### Evaluation of linearity of dosage–exposure relationship

The dose proportionality was analyzed via a power model [21, 22]. The model was fitted as a linear mixed-effects model that included a random subject effect (Eq 1):

$$\text{ln}\left({\text{PK}}_{ij}\right)=\mu +{\text{a}}_{j}+\beta \;\cdot \text{ ln}\left({\text{Dose}}_{i}\right)+\varepsilon_{ij}$$ (1)

where PK_*ij*_ is the AUC_*0–last*_ or *C*_max_ at dose i (*i* = 1, 2, 3) in the subjects j (*j* = 1, 2, …, *n*), μ is the overall mean, a_*j*_ is a random subject effect that describes the individual difference for subject *j* and is assumed to be normally distributed around mean 0 with variance σ_a_^2^, Dose_*i*_ is the administered dose (g) of the test drug, ε_*ij*_ represents random error with mean 0 and variance σ^2^. β is a parameter to be used for dose proportionality evaluation. Inferences were made based on the theoretical β of 1 and on confidence limits of 0.8 and 1.25. Evaluation of linearity of the dosage-exposure relationship was conducted with Phoenix WinNonlin and SAS 9.2 (SAS Institute, Inc., Cary, NC).

## Results

### Registered subjects

Twenty one subjects were screened and enrolled for the present study. Table 2 shows demographics of the 21 subjects in each group. One and two subjects were discontinued in this study after first or second dosing since the visit schedule was not able to be adjusted. However the data from first dosing were included in the pharmacokinetic analysis. No adverse effects were observed in subjects treated with rikkunshito.

**Table 2:** Clinical and demographic characteristics of study participants. BMI; body mass index. Data represent the median (minimum–maximum).

Group	*n*	Age	Height (cm)	Weight (kg)	BMI (kg/m^2^)	Male/Female
A	7	25 (21–35)	157.5 (152.7–178.9)	51.4 (44.5–79.3)	22.0 (18.6–24.8)	2/5
B	7	25 (21–40)	169.5 (155.5–180.5)	57.8 (46.4–81.4)	21.2 (19.2–25.0)	5/2
C	7	26 (24–31)	171.9 (145.5–183.6)	64.5 (42.1–84.1)	23.0 (18.5–25.0)	6/1

### Plasma concentrations of rikkunshito ingredients

The presence of 18 of 32 ingredients tested were determined in plasma samples from four subjects after oral administration of rikkunshito (Table 3). Among these ingredients, the plasma concentration of 18β-glycyrrhetinic acid was greatest, with *C*_max_ of 58,200 pg/mL 8 h after administration. The ingredient showing the next highest *C*_max_ was atractylodin at 1380 pg/ml 1 h after administration. It was followed by oleanolic acid showing 1120 pg/ml 8 h after administration. Other ingredients that were quantifiable at no lesser than two time points after rikkunshito administration were pachymic acid, liquiritin apioside, liquiritin, isoliquiritigenin, glycycoumarin, glycyrrhetinic acid 3-*O*-glucuronide, nobiletin, 3,3′,4′,5,6,7,8-heptamethoxyflavone (heptamethoxyflavone), and naringenin. For pachymic acid, 18β-glycyrrhetinic acid, atractylodin, and naringenin, some contaminating peaks were detected in plasma even before rikkunshito administration, which were thought to be derived from food; however, these peaks were found in only one of four subjects or were at lower than approximately one-half of the concentrations observed after rikkunshito administration.

**Table 3:** Quantification of rikkunshito ingredients in plasma. BQL; below the quantification limit. Plasma concentration of [6]-gingerol, [8]-gingerol, [10]-gingerol, [6]-shogaol, [8]-shogaol, ginsenoside Rb_2_, ginsenoside Re, ginsenoside Rf, ginsenoside Rg_1_, ginsenoside Rg_2_, hesperidin, hesperetin, narirutin, and PTH-15 were BQL at all time points. Each value represents mean ± standard deviation (S.D.) (*n* = 4).

Compound	Plasma concentration (pg/mL)					
	0 h	0.5 h	1 h	2 h	4 h	8 h
Ginsenoside Rb_1_	BQL	BQL	BQL	BQL	175 ± 118	BQL
Ginsenoside Rc	BQL	BQL	BQL	BQL	30.3 ± 60.5	BQL
Ginsenoside Rd	BQL	BQL	30.8 ± 61.5	BQL	BQL	BQL
Pachymic acid	140 ± 184	126 ± 185	127 ± 178	205 ± 76.5	90.5 ± 181	103 ± 129
Liquiritin apioside	BQL	340 ± 91.8	473 ± 94.2	420 ± 163	204 ± 139	BQL
Liquiritin	BQL	1020 ± 131	841 ± 94.3	403 ± 91.3	114 ± 39.2	BQL
Isoliquiritigenin	BQL	15.3 ± 18.0	BQL	17.1 ± 34.3	BQL	BQL
Glycycoumarin	BQL	9.23 ± 18.5	7.15 ± 14.3	6.93 ± 13.9	BQL	BQL
18β-Glycyrrhetinic acid	205 ± 329	970 ± 116	776 ± 247	8200 ± 11400	42200 ± 37100	58200 ± 8420
Glycyrrhetic acid 3-*O*-glucuronide	BQL	BQL	44.8 ± 89.5	244 ± 73.5	327 ± 65.6	247 ± 62.2
Atractylodin	328 ± 655	1190 ± 913	1380 ± 1150	863 ± 1010	1140 ± 899	295 ± 590
Nobiletin	BQL	47.0 ± 47.4	28.2 ± 38.9	5.45 ± 10.9	BQL	BQL
Tangeretin	BQL	5.73 ± 11.5	BQL	BQL	BQL	BQL
Heptamethoxyflavone	BQL	171 ± 180	253 ± 301	157 ± 187	56.9 ± 82.1	6.13 ± 12.3
Synephrine	BQL	29.0 ± 58.0	BQL	BQL	BQL	BQL
Naringin	BQL	BQL	59.0 ± 68.4	BQL	BQL	BQL
Naringenin	378 ± 755	35.8 ± 71.5	BQL	81.3 ± 105	71.5 ± 83.1	BQL
Oleanolic acid	BQL	BQL	BQL	198 ± 395	903 ± 983	1120 ± 890

Enzymatic treatment of plasma samples with β-glucuronidase resulted in markedly increased concentrations of four ingredients: glycycoumarin, hesperetin, isoliquiritigenin, and naringenin compared to the respective pretreatment concentrations (Table 4). Unchanged glycycoumarin was detected in only one subject before enzyme treatment at low concentrations at approximately 30 pg/ml at any time point; however, the concentration increased to 2340 pg/ml at the 30-min time point after enzyme treatment.

**Table 4:** Quantification of rikkunshito ingredients in enzyme-treated plasma. BQL; below the quantification limit. Each value represents mean ± S.D. (*n* = 4).

Compound	Plasma concentration (pg/mL)					
	0 h	0.5 h	1 h	2 h	4 h	8 h
Isoliquiritigenin	BQL	87.2 ± 22.1	55.5 ± 18.7	60.6 ± 77.4	69.8 ± 33.5	23.8 ± 35.4
Glycycoumarin	BQL	2340 ± 848	987 ± 360	328 ± 183	371 ± 113	222 ± 134
Hesperetin	BQL	BQL	BQL	227 ± 264	799 ± 408	307 ± 289
Naringenin	BQL	311 ± 211	261 ± 188	351 ± 311	861 ± 749	156 ± 234

The plasma concentration of unchanged hesperetin before the enzyme treatment was below the quantification limit (BQL) at any time point. After enzymatic treatment, the concentration was in the quantifiable range in plasma samples obtained 2, 4, and 8 h after rikkunshito administration, of which the 4-h plasma showed *C*_max_ of 799 pg/ml. The plasma concentration of unchanged isoliquiritigenin without enzymatic treatment was BQL in two of four subjects at all time points and was 15.3 pg/ml at the 30-min time point. After enzyme treatment, the concentration was in the quantifiable range in four subjects, and *C*_max_ was 87.2 pg/ml at the 30-min time point.

**Fig 2:**
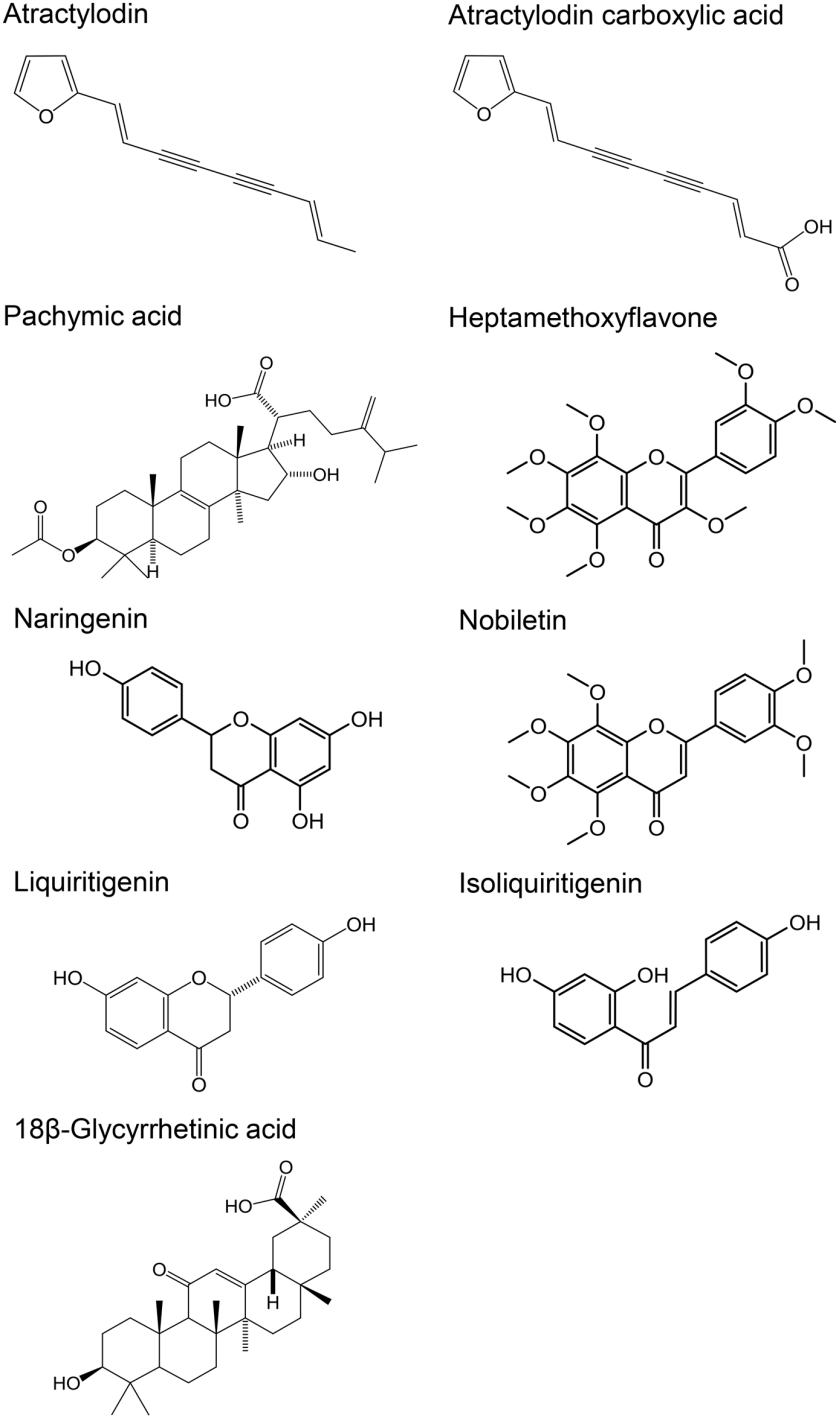
Chemical structures of nine ingredients derived from rikkunshito

**Fig 3:**
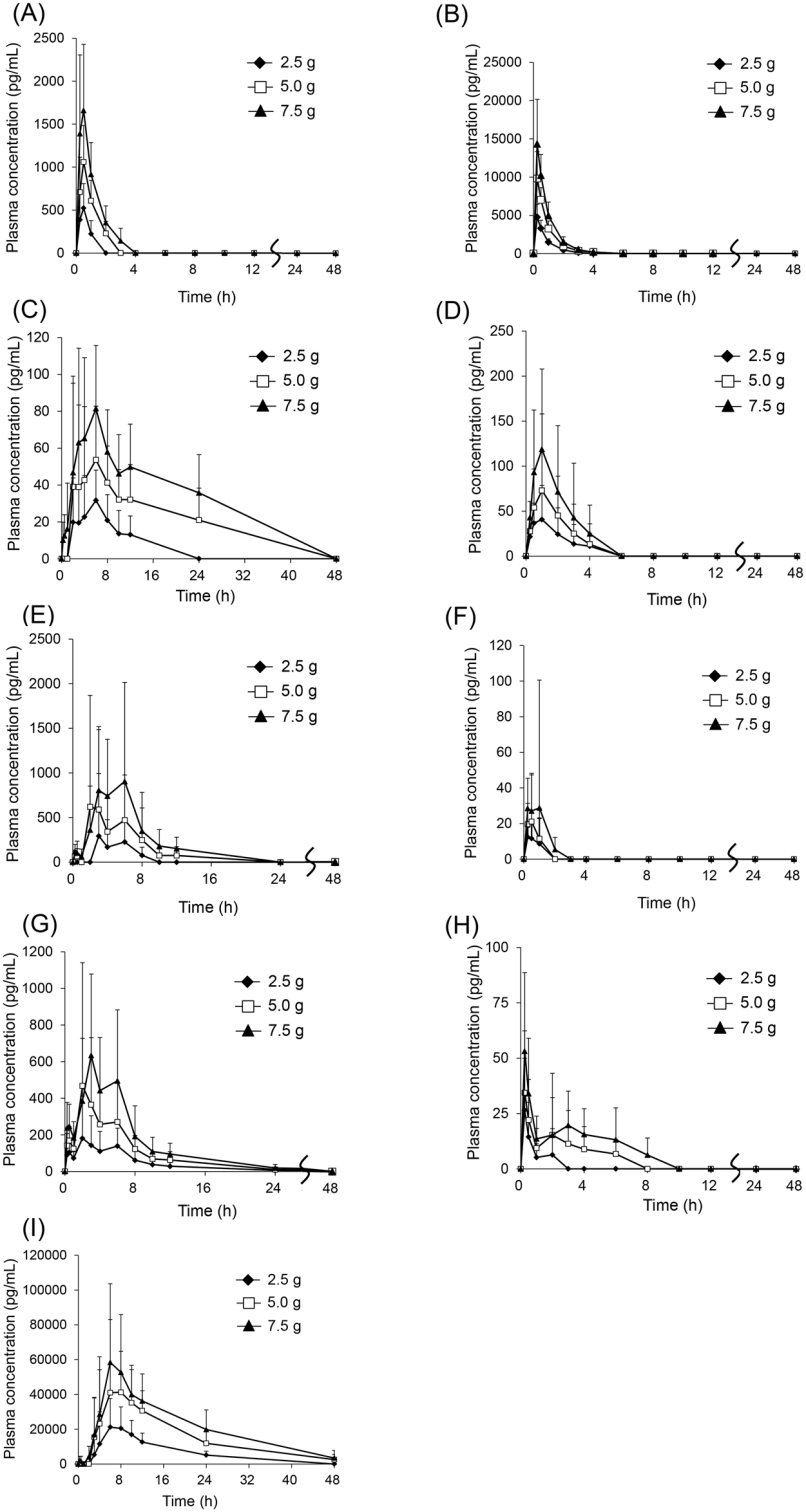
Plasma concentrations of nine ingredients derived from rikkunshito A; atractylodin, B; atractylodin carboxylic acid, C; pachymic acid, D; heptamethoxyflavone, E; naringenin, F; nobiletin, G; liquiritigenin, H; isoliquiritigenin, I; 18β-glycyrrhetinic acid. Blood samples were collected at 0 (beginning of the study), 0.25, 0.5, 1, 2, 3, 4, 6, 8, 10, 12, 24, and 48 h after administration of rikkunshito (2.5, 5.0, or 7.5 g/day). Each value represents mean ± S.D. (*n* = 19–21).

Among the ingredients detected in their unchanged forms by the exploratory study in plasma samples from four subjects, eight ingredients closely involved in the efficacy and adverse effects of rikkunshito were analyzed in 21 subjects. The linear range for the assay of atractylodin, pachymic acid, heptamethoxyflavone, naringenin, nobiletin, liquiritigenin, isoliquiritigenin, and 18β-glycyrrhetinic acid were 200–20,000, 10–1,000, 4–400, 50–5,000, 4–400, 2–200, 2–200, and 800–80,000 pg/mL, respectively. The intra-assay and inter-assay precision (% coefficient of variation) of quality control samples were ≤14.9%. The validation items and results are summarized in S8 Table. The structures and time profiles of changes in plasma concentrations of the eight ingredients and atractylodin metabolite are shown in Figs 2 or 3. The pharmacokinetic parameters of the compounds are shown in Table 5. 18β-glycyrrhetinic acid showed the highest *C*_max_, at a dose of 7.5 g, followed by atractylodin carboxylic acid, naringenin, liquiritigenin, heptamethoxyflavone, pachymic acid, isoliquiritigenin, and nobiletin. When the *t*_max_ values of the ingredients at a dose of 7.5 g were compared, it was found that atractylodin carboxylic acid, isoliquiritigenin, nobiletin, atractylodin, and heptamethoxyflavone were all rapid-acting, with values of 1 h or shorter, while liquiritigenin, naringenin, pachymic acid, and 18β-glycyrrhetinic acid showed values exceeding 3 h. When *t*_1/2_ values of the components at a dose of 7.5 g were compared, atractylodin carboxylic acid, atractylodin, heptamethoxyflavone, nobiletin, isoliquiritigenin, liquiritigenin, and naringenin had values < 10 h, while 18β-glycyrrhetinic acid, and pachymic acid had values exceeding 10 h. The plasma concentrations of atractylodin and pachymic acid just before the administration were BQL in all subjects, whereas peaks for other compounds (atractylodin carboxylic acid, heptamethoxyflavone, liquiritigenin, isoliquiritigenin, nobiletin, naringenin, and 18β-glycyrrhetinic acid) were detected in some subjects. The washout period of 4 weeks used in this study is sufficiently longer than five half-lives of any ingredient analyzed. Accordingly, we inferred that these ingredient peaks observed in preadministration plasma samples were food-derived rather than carry-over.

**Table 5:** Pharmacokinetic parameters of rikkunshito ingredients. -: *t*_1/2_ was not calculated for all subjects.

Parameter	2.5 g (*n* = 21)	5.0 g (*n* = 19)	7.5 g (*n* = 19)
	Geometric mean (95% CI)	Geometric mean (95% CI)	Geometric mean (95% CI)
Atractylodin			
*C*_max_, pg/mL	531 (425–662)	1020 (837–1250)	1570 (1210–2040)
AUC_*0-last*_, pg·h/mL	318 (224–453)	1060 (860–1300)	1760 (1290–2390)
*t*_1/2_, h	-	0.746 (0.470–1.18)	0.888 (0.687–1.15)
Median *t*_max_, h (range)	0.500 (0.250–2.00)	0.500 (0.250–1.00)	0.500 (0.250–1.00)
Atractylodin carboxylic acid			
*C*_max_, pg/mL	4450 (3700–5350)	9570 (8230–11100)	14300 (12200–16800)
AUC_*0-last*_, pg·h/mL	3910 (3470–4390)	8740 (7720–9900)	12700 (11100–14600)
*t*_1/2_, h	0.580 (0.510–0.650)	0.637 (0.550–0.730)	0.663 (0.560–0.790)
Median *t*_max_, h (range)	0.250 (0.250–1.00)	0.250 (0.250–0.500)	0.250 (0.250–0.500)
Pachymic acid			
*C*_max_, pg/mL	36.3 (28.2–46.8)	61.2 (46.0–81.5)	91.0 (71.8–115)
AUC_*0-last*_, pg·h/mL	277 (189–407)	716 (539–953)	1210 (882–1650)
*t*_1/2_, h	-	16.2 (7.83–33.6)	20.5 (14.4–29.2)
Median *t*_max_, h (range)	6.00 (2.00–8.00)	6.00 (2.00–23.6)	6.00 (2.00–11.5)
Heptamethoxyflavone			
*C*_max_, pg/mL	38.8 (29.8–50.6)	58.2 (41.2–82.1)	105 (75.6–144)
AUC_*0-last*_, pg·h/mL	84.9 (51.9–139)	124 (82.9–184)	225 (157–322)
*t*_1/2_, h	1.92 (1.29–2.88)	1.50 (1.12–2.02)	1.51 (1.15–1.98)
Median *t*_max_, h (range)	0.500 (0.250–2.02)	1.00 (0.250–2.03)	1.00 (0.250–1.02)
Naringenin			
*C*_max_, pg/mL	376 (240–590)	878 (586–1320)	1150 (802–1650)
AUC_*0-last*_, pg·h/mL	1650 (926–2940)	3320 (2310–4780)	4630 (2930–7320)
*t*_1/2_, h	-	4.46 (2.40–8.28)	5.38 (3.31–8.76)
Median *t*_max_, h (range)	5.83 (0.250–23.7)	4.00 (2.00–8.00)	4.00 (2.00–6.00)
Nobiletin			
*C*_max_, pg/mL	15.6 (12.1–20.1)	19.1 (13.5–27.1)	35.9 (24.6–52.5)
AUC_*0-last*_, pg·h/mL	17.7 (8.76–35.8)	23.8 (12.0–47.1)	35.7 (20.4–62.7)
*t*_1/2_, h	-	3.47 (1.14–10.5)	2.37 (0.627–8.96)
Median *t*_max_, h (range)	0.267 (0.250–11.5)	0.267 (0.250–10.0)	0.283 (0.250–3.00)
Liquiritigenin			
*C*_max_, pg/mL	235 (175–315)	540 (379–769)	800 (672–952)
AUC_*0-last*_, pg·h/mL	1180 (931–1500)	2560 (2040–3210)	4040 (3260–5010)
*t*_1/2_, h	-	4.42 (3.58–5.46)	5.13 (4.00–6.57)
Median *t*_max_, h (range)	4.00 (0.500–6.02)	3.00 (0.500–8.00)	3.02 (2.00–6.00)
Isoliquiritigenin			
*C*_max_, pg/mL	20.5 (14.3–29.3)	32.1 (22.9–45.0)	42.8 (30.4–60.3)
AUC_*0-last*_, pg·h/mL	30.7 (19.3–48.8)	76.6 (55.9–105)	122 (88.2–168)
*t*_1/2_, h	-	4.12 (1.85–9.18)	3.26 (1.69–6.31)
Median *t*_max_, h (range)	0.250 (0.233–4.00)	0.250 (0.233–6.02)	0.250 (0.250–4.00)
18β-Glycyrrhetinic acid			
*C*_max_, pg/mL	23000 (17300–30700)	44200 (31500–62100)	55600 (39600–78100)
AUC_*0-last*_, pg·h/mL	292000 (242000–352000)	638000 (499000–816000)	832000 (628000–1100000)
*t*_1/2_, h	-	8.94 (6.68–12.0)	11.7 (8.90–15.3)
Median *t*_max_, h (range)	8.00 (4.00–23.6)	8.00 (3.00–47.5)	8.00 (5.98–23.7)

### Urinary concentrations of rikkunshito ingredients

We attempted to detect 32 ingredients in urine samples after rikkunshito administration to four subjects, and detected 21 ingredients (Table 6). Liquiritin showed the highest urine concentration at 7,790 ng followed by liquiritin apioside at 4,330 ng at 0–4 h postadministration. Besides, hesperetin showed the highest concentration at 5,160 ng, followed by naringenin at 2,380 ng at 4–8 h postadministration. Some ingredient peaks were found in urine samples collected before rikkunshito administration, similar to that with plasma samples. However, the concentrations were less than one-fourth of those in postadministration samples, except for narirutin, which was found only in urine samples collected before administration.

**Table 6:** Quantification of rikkunshito ingredients in urine. BQL; below the quantification limit. Amount of ingredients of [10]-gingerol, ginsenoside Rb_1_, ginsenoside Rc, ginsenoside Rd, ginsenoside Rg_2_, pachymic acid, nobiletin, tangeretin, heptamethoxyflavone, PTH-15, and oleanolic acid in urine were BQL at all time points. Each value represents mean ± S.D. (*n* = 4).

Compound	Amount (ng)		
	Pre	0–4 h	4–8 h
[6]-Gingerol	18.6 ± 26.2	602 ± 552	35.0 ± 57.1
[8]-Gingerol	BQL	8.97 ± 17.9	BQL
[6]-Shogaol	16.3 ± 26.4	459 ± 283	15.4 ± 30.7
[8]-Shogaol	BQL	9.00 ± 18.0	BQL
Ginsenoside Rb_2_	BQL	202 ± 202	84.6 ± 90.1
Ginsenoside Re	BQL	BQL	30.6 ± 61.2
Ginsenoside Rf	BQL	48.2 ± 64.4	23.0 ± 20.4
Ginsenoside Rg_1_	BQL	620 ± 331	638 ± 319
Liquiritin apioside	BQL	4330 ± 1530	2370 ± 1080
Liquiritin	15.7 ± 19.0	7790 ± 1510	1900 ± 945
Isoliquiritigenin	0.688 ± 1.38	741 ± 393	1010 ± 451
Glycycoumarin	4.61 ± 4.20	2300 ± 1150	1600 ± 886
18β-Glycyrrhetinic acid	BQL	BQL	93.0 ± 60.7
Glycyrrhetic acid 3-*O*-glucuronide	BQL	BQL	6.94 ± 13.9
Atractylodin	BQL	75.8 ± 152	BQL
Hesperidin	28.9 ± 57.8	184 ± 60.5	68.6 ± 47.9
Hesperetin	213 ± 390	462 ± 418	5160 ± 3380
Narirutin	9.26 ± 18.5	BQL	BQL
Synephrine	24.2 ± 28.8	2150 ± 296	798 ± 282
Naringin	70.7 ± 69.2	1030 ± 313	598 ± 329
Naringenin	BQL	BQL	2380 ± 2940

Urine concentrations of [6]-gingerol, [8]-gingerol, [6]-shogaol, [8]-shogaol, glycycoumarin, hesperetin, and isoliquiritigenin markedly increased after treatment of urine with β-glucuronidase compared with those before treatment (Table 7). Among these ingredients, urine concentrations of [6]-gingerol, [6]-shogaol, [8]-shogaol, and hesperetin showed increases of more than 10-fold following treatment.

**Table 7:** Quantification of rikkunshito ingredients in enzyme-treated urine. BQL; below the quantification limit. Because a calibration curve could not be constructed, owing to a matrix effect (urine treated with β-glucuronidase), naringenin was not analyzed. Each value represents mean ± S.D. (*n* = 4).

Compound	Amount (ng)		
	Pre	0–4 h	4–8 h
[6]-Gingerol	278 ± 261	8170 ± 5630	821 ± 654
[8]-Gingerol	2.73 ± 5.46	49.5 ± 78.3	BQL
[6]-Shogaol	109 ± 160	4980 ± 1480	392 ± 310
[8]-Shogaol	3.73 ± 7.46	239 ± 62.5	BQL
Isoliquiritigenin	16.0 ± 17.4	3060 ± 348	5630 ± 2210
Glycycoumarin	22.8 ± 19.7	7010 ± 2840	3260 ± 1580
Hesperetin	4290 ± 7690	7130 ± 9290	56750 ± 37530

### Contents of 16 ingredients in rikkunshito extracts

The contents of 15 ingredients in 1 g of rikkunshito were hesperidin, 3750 μg; glycyrrhizic acid, 1370 μg; narirutin, 932 μg; liquiritin, 801 μg; liquiritin apioside, 697 μg; isoliquiritin, 101 μg; isoliquiritin apioside, 85.2 μg; liquiritigenin, 79.8 μg; pachymic acid, 67.5 μg; atractylodin, 56.3 μg; heptamethoxyflavone, 23.4 μg; nobiletin, 17.4 μg; isoliquiritigenin, 9.85 μg; naringenin, 3.95 μg; and 18β-glycyrrhetinic acid, 1.84 μg. The content of naringin was not detected.

### Dose linearity test by power regression analysis for C_max_ and AUC_0–last_ of nine ingredients derived from rikkunshito

**Fig 4:**
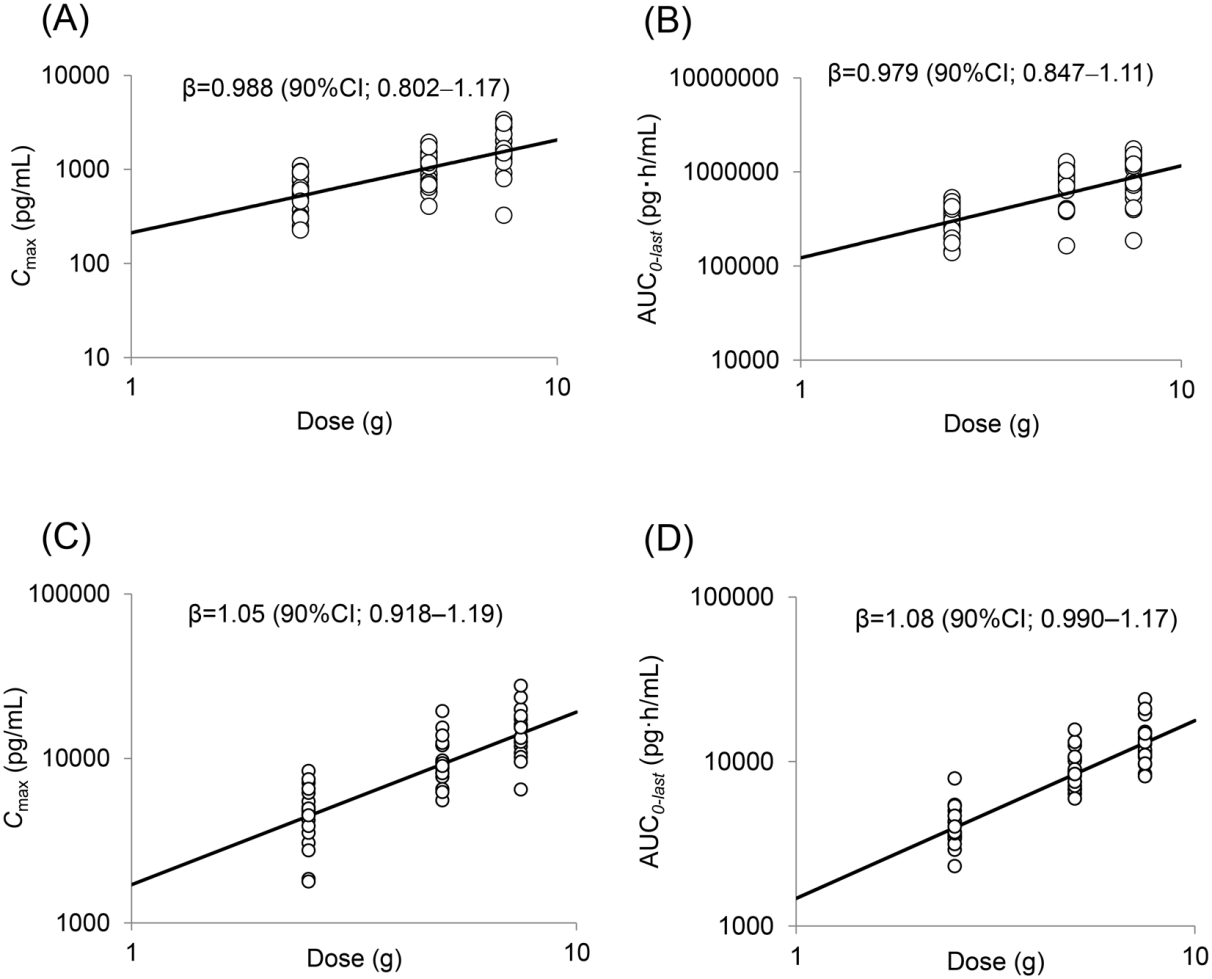
Relations between dosage and *C*_max_ or AUC_*0–last*_ A; atractylodin, B; 18β-glycyrrhetinic acid, C, D; atractylodin carboxylic acid. A power regression model was fitted to a mixed-effect model for evaluation of linearity. CI; confidence interval.

The dose proportionality of *C*_max_ and AUC_*0–last*_ is graphically displayed in Fig 4. The system was considered to be linear when CI_lower_ ≥ 0.8 and CI_upper_ ≤ 1.25. The β value (90% CI) of atractylodin and atractylodin carboxylic acid for *C*_max_ and atractylodin carboxylic acid and 18β-glycyrrhetinic acid for AUC_*0–last*_ were 0.988 (0.802–1.17), 1.05 (0.918–1.19), 1.08 (0.990–1.17), and 0.979 (0.847–1.11), respectively. These results suggested that atractylodin and atractylodin carboxylic acid for *C*_max_ and atractylodin carboxylic acid and 18β-glycyrrhetinic acid for AUC_*0–last*_ were linear within the dose range of 2.5–7.5 g/day of rikkunshito. However, the 90% CI of the β value of the other parameters did not include 1, and confidence limits were out of the range of 0.8–1.25 (S9 Table).

## Discussion

To the best of our knowledge, for the first time this study revealed the presence of several active ingredients and their metabolites, known to act as ghrelin enhancers, in plasma and urine of humans who orally received rikkunshito. We also investigated changes in plasma concentration of these ingredients and analyzed linearity, based on the pharmacokinetic parameters obtained in this study.

Recent basic studies have identified likely ingredients involved in various ghrelin-associated actions of rikkunshito, including hesperidin and flavonoids such as heptamethoxyflavone, in promotion of ghrelin secretion via 5-HT_2B_ and 5-HT_2C_R antagonism [14, 23], atractylodin in enhancement of ghrelin receptor signaling [1], and pachymic acid in extension of the effect of active-form ghrelin via inhibition of ghrelin metabolism [17]. Another study had shown that oral administration of hesperidin, heptamethoxyflavone, and isoliquiritigenin in a cisplatin-induced anorexia model suppressed the decrease of the acylated ghrelin level in blood in a concentration-dependent manner [14]. In the present study, pharmacokinetic analysis was conducted with particular focus on the ingredients involved in the ghrelin enhancer activity of rikkunshito.

First, we constructed a simultaneous multicomponent analysis system for 32 representative ingredients of rikkunshito and analyzed their plasma concentrations after rikkunshito administration in humans; among them, 18 ingredients were detectable in human plasma. However, no ingredient was detected at a high concentration sufficient to suggest a single ingredient that can explain the pharmacological mechanism underlying the effect of rikkunshito. In particular, hesperetin (aglycone of hesperidin) and isoliquiritigenin (aglycone of isoliquiritin), which inhibit 5-HT_2B_R and 5-HT_2C_R and are considered to be strongly involved in the promotion of ghrelin secretion [14], were barely present in plasma. This result indicates that the sensitivity of the analysis needs to be further increased when these ingredients are to be analyzed in future studies. In plasma and urine samples treated with β-glucuronidase, target ingredients other than hesperetin and isoliquiritigenin were detected. According to this result, it became clear that some active ingredients were mainly present in their conjugate forms in plasma.

Based on the plasma concentrations of ingredients measured in the exploratory pharmacokinetic study and their contributions to the pharmacological effect or adverse effects, target ingredients for analysis were narrowed to eight ingredients, and a randomized crossover study was conducted with these eight ingredients. As a result, the *C*_max_ of atractylodin, which enhances ghrelin signaling [1], was 1570 pg/ml after administration of 7.5 g of rikkunshito and was the highest value among eight ingredients measured, except for 18β-glycyrrhetinic acid.

Reports on pharmacokinetics of compounds structurally related to atractylodin are few. This study is the first to show the absorption of atractylodin into plasma after oral administration of rikkunshito in humans. Because *t*_max_ of atractylodin is as short as 0.5 h and *t*_1/2_ is approximately 1 h after 7.5 g rikkunshito administration, it may be involved in the orexigenic effect that occurs in the early period after rikkunshito administration. However, comparing results from *in vitro* experiments with ghrelin-expressing HEK293 cells [1], the plasma concentration of atractylodin may need to be much higher to show activity. For atractylodin, we also quantified metabolite having ghrelin signal enhancement activity similar to that of the unchanged form (S1 Fig). *C*_max_ of the metabolite was 5.7–31.5-fold of that of the unchanged form after administration of 7.5 g rikkunshito. Accordingly, we inferred the ghrelin signal enhancement activity of rikkunshito to be mediated by the unchanged form and the active metabolite of atractylodin in combination. Ghrelin receptors, a target of atractylodin, are localized at vagus nerve endings in gastric mucosa. We discovered that atractylodin is stable in a solution with the same pH as that in the stomach (S10 Table). Thus, the pharmacological effect of atractylodin may be partly attributed to its direct action in the digestive tract.

Heptamethoxyflavone, nobiletin, and naringenin are polymethoxyflavones lacking sugar moieties. These flavones have the most potent 5-HT_2B_R antagonistic activity among all rikkunshito ingredients [14]. The *t*_max_ values of nobiletin and heptamethoxyflavone were between 15 min and 1 h. Both heptamethoxyflavone and nobiletin were then eliminated from plasma without passing through the enterohepatic circulation, and their respective *t*_1/2_ were 1.51 and 2.37 h after 7.5 g rikkunshito administration. In contrast, changes in plasma concentration of naringenin showed bimodality, with *t*_max_ of 2–3 h or 6 h and *t*_1/2_ of 5.38 h after 7.5 g rikkunshito administration. These ingredients are similar in structure, but their *t*_max_ and *t*_1/2_ values are different. Accordingly, we speculated that these ingredients were acting successively on active sites after rikkunshito administration.

Isoliquiritigenin, a flavonoid, inhibits 5-HT_2B_R and 5-HT_2C_R activities [14]. The *C*_max_ of isoliquiritigenin was extremely low at approximately 42.8 pg/ml after 7.5 g rikkunshito administration, and *t*_1/2_ of the first phase was very fast; however, changes in its plasma concentration showed bimodality. Isoliquiritigenin in rikkunshito is mainly present as a glycoside, isoliquiritin. Glycosides generally become absorbable after sugar moieties are removed by enterobacteria and enter the blood circulation. Given that the *t*_max_ of the first peak of isoliquiritigenin detected in plasma was fast, it was thought that the peak was derived not from glycosides but from the aglycone in rikkunshito. It is not currently clear whether the second peak is caused by enterohepatic circulation or by the timing of glycoside absorption, and the question needs further study.

Although absorption of isoliquiritigenin and hesperetin into the body was evident from the results obtained in this study, they were present as conjugate forms in the blood circulation and the concentrations of their unchanged forms were very low to explain the pharmacological effect. In general, it is considered that the pharmacological action of a compound becomes weaker after its conversion to a glycoside form. The largest amount of an ingredient contained in rikkunshito extract is a glycoside form of hesperetin, hesperidin; moreover, the combined amount of isoliquiritigenin, isoliquiritin, and isoliquiritin apioside, which are glycoside forms of isoliquiritigenin, is 196 μg in 1 g of rikkunshito. Oral administration of hesperidin or isoliquiritigenin at a dose of 4.0 mg/kg inhibits a decrease in plasma ghrelin concentration [14] and also improves decreased appetite [15] following cisplatin administration. These findings suggest the possibility that the conjugate form itself exerts the activity or that the conjugate form serves as a carrier from plasma into the target organ.

Because 5-HT_2B_R is located in the mucosa of the intestinal tract, it is possible that some ingredients directly act on the receptor during absorption through the intestinal tract. However, 5-HT_2C_R is localized in the central nervous system; this means that ingredients should pass through blood brain barrier (BBB) to demonstrate their pharmacological effects. In an *in vitro* BBB model system, hesperetin was detected on the basal side in a state of unchanged form when hesperetin glucuronide was added on the apical side [24]. Thus, it is possible that ingredient conjugates in plasma exist as active free forms in a target organ. It is difficult to confirm whether an active-form ingredient is indeed present in the human brain. However, it is essential to investigate whether active ingredients reach the brain to elucidate the mechanism of the pharmacological effects of rikkunshito. Thus, the presence of the active-form ingredients in the brain should be verified by experiments in animals or BBB model cells.

Ghrelin exerts a physiological activity as acyl ghrelin, which is metabolized by esterase in the body to des-acyl ghrelin, which lacks the activity. Therefore, inhibition of ghrelin metabolism also represents a possible therapeutic target for anorexia. Pachymic acid has been reported to inhibit butyrylcholinesterase and is thought to play a role in the orexigenic effect of rikkunshito [17]. Although the *C*_max_ of pachymic acid was very low at 91.0 pg/ml following 7.5 g rikkunshito administration, its elimination was slow with a *t*_1/2_ of 20.5 h and was characterized by two elimination phases. This ingredient is not a glycoside, but had a *t*_max_ of 6 h, which was longer than the *t*_max_ of other nonglycoside ingredients. Accordingly, we predicted that pachymic acid could remain in the body for a prolonged period, albeit at low concentration, and contribute to drug efficacy for a long period. It is conceivable that the plasma concentration of pachymic acid increases with continuous administration of rikkunshito.

*G*. *radix* is a constituent crude drug in several Kampo formulations and has been reported to have several pharmacological activities [25–27]. 18β-glycyrrhetinic acid, a *G*. *radix*-derived ingredient, has a *t*_max_ of 8 h, which is very slow, and we speculate that the peak of 18β-glycyrrhetinic acid was derived from a glycoside form. In rare cases, *G*. *radix* triggers hypokalemia in humans [28], and a 18β-glycyrrhetinic acid-related ingredient has been suggested as a possible cause [29]. Rikkunshito also contains glycyrrhiza; therefore, the possibility of triggering of hypokalemia by rikkunshito cannot be ignored. Although the frequency of developing adverse effect by rikkunshito is currently under investigation, the occurrence rate of hypokalemia with rikkunshito may be lower than that with another Kampo medicine, yokukansan, which contains more glycyrrhizic acid than rikkunshito and has been reported to cause hypokalemia with frequency 1.3% [30].

To determine the pharmacokinetic characteristics of each ingredient, we evaluated the linearity between an administered dose and *C*_max_ or AUC_*0–last*_. The results suggested that these parameters did not show linearity for the ingredients except atractylodin, atractylodin carboxylic acid, and 18β-glycyrrhetinic acid. This observation may be accounted for by the high variation between subjects for almost all ingredients. Possible reasons for this variation are that many of the ingredients were metabolized by enterobacteria during absorption and that some ingredients underwent conjugation reactions by metabolic enzymes in the intestinal tract. Another possible reason is that plasma concentrations for most ingredients analyzed in this study were near the lower limits of their quantifiable ranges.

Given that a Kampo formulation is a multi-component drug, its effect is expressed as a complex combination of pharmacological and pharmacokinetic properties of individual ingredients metabolized and absorbed into the body at different rates. Multiple ingredients of rikkunshito were also absorbed into the body and showed various profiles in plasma (S11 Table). The results of this study do not support the notion that the pharmacological effect of rikkunshito is mediated by a single ingredient, but suggest that the effect is mediated by multiple ingredients acting successively on a target molecule. Rikkunshito contains several ingredients with analogous structures, such as those containing the flavonoid skeleton. These similar ingredients are highly likely to produce the same compound as a metabolite, and for this reason, it may be difficult to explain the drug action based on their contents in the formulation.

## Conclusion

For the first time, we measured plasma concentrations of active ingredients after rikkunshito administration and calculated their pharmacokinetic parameters. Although ingredients that potentially mediate the activity of rikkunshito were detected in plasma, no ingredient that could independently explain the activity was detected. The effect of rikkunshito is suggested to be exerted through a synergistic action of various ingredients and local direct action in the stomach. The results obtained in this study will be very useful for elucidating the mechanism of action of rikkunshito and also provide beneficial information to medical personnel who use rikkunshito in clinical settings.

## Supporting Information

S1 CONSORT Checklist CONSORT 2010 checklist of information to include when reporting a randomized trial. (DOC)

S1 Protocol Quantitative trial protocol. (DOC)

S1 Appendix Methods of analysis of rikkunshito ingredients. (DOCX)

S1 Fig Effect of atractylodin and atractylodin carboxylic acid on ghrelin/GHS-R binding activity. Radioligand binding was performed using GSH-R-expressing cells (*n* = 3).
(TIF)

S1 Table Typical 32 ingredients included in rikkunshito. (DOCX)

S2 Table Methods of LC-MS/MS for analysis of plasma and urine samples: Ion parameters of rikkunshito ingredients and internal standards. (DOCX)

S3 Table Methods of LC-MS/MS for analysis of plasma and urine samples: HPLC conditions for analyzing rikkunshito ingredients. (DOCX)

S4 Table Methods of LC-MS/MS for analysis of plasma samples: Ion parameters of 9 ingredients derived from rikkunshito and internal standards. (DOCX)

S5 Table Methods of LC-MS/MS for analysis of plasma samples: Conditions of HPLC conditions for analyzing 9 ingredients derived from rikkunshito. (DOCX)

S6 Table Methods of LC-MS/MS for analysis of rikkunshito formulation: Ion parameters of rikkunshito ingredients, and internal standards. (DOCX)

S7 Table Methods of HPLC and LC-MS/MS for analysis of rikkunshito formulation: Conditions of HPLC conditions for analyzing rikkunshito ingredients. (DOCX)

S8 Table Validation items and results of 8 ingredients derived from rikkunshito. (DOCX)

S9 Table Dose proportionality test using power model. (DOCX)

S10 Table Stability of 32 ingredients in gastric pH solution. (DOCX)

S11 Table Formation of ingredients derived from rikkunshito in formulation, blood, and urine. (DOCX)
