# Supplementary material for: Pharmacokinetic Profiles of Active Ingredients and Its Metabolites Derived from Rikkunshito, a Ghrelin Enhancer, in Healthy Japanese Volunteers: A Cross-Over, Randomized Study
Source: PLoS One. 2015 Jul 17;10(7):e0133159. doi: 10.1371/journal.pone.0133159 (PMC4506051; doi:10.1371/journal.pone.0133159)
Supplement: S1 Protocol — (DOC) [file pone.0133159.s002.doc]

| TJ-43-4-2 |
| --- |
| Ver. 1.1 |
| Prepared on June 2, 2014 |

Pharmacokinetic Study of Single Administration of Rikkunshito (TJ-43) in Healthy Volunteers

Post-Marketing Clinical Study Protocol

Ver. 1.1

Prepared on June 2, 2014

| Sponsor | Tsumura & Co., Kampo Research Planning Department |
| --- | --- |
|  | 2-17-11 Akasaka Minato-ku, Tokyo, 107-8521, Japan  TEL. +813-6361-7184 (direct) |

**Table of Contents**

**1** **STUDY SYNOPSIS 1**

**1.1** **Title 1**

**1.2** **Study Objective 1**

**1.3** **Endpoints 1**

**1.4** **Study Population 1**

1.4.1 Subjects 1

1.4.2 Target Sample Size 1

1.4.3 Inclusion Criteria 1

1.4.4 Exclusion Criteria 1

**1.5** **Study Design 2**

**1.6** **Dosage and Administration 2**

**1.7** **Schedule of Time And Events 2**

**1.8** **Other Factors for Control 2**

**1.9** **Study Period 2**

**1.10** **Testing Institution 2**

**1.11** **Sponsor 2**

**2** **BACKGROUND 3**

**3** **STUDY OBJECTIVE 4**

**3.1** **Study Objective 4**

**3.2** **Type of Study 4**

**4** **STUDY POPULATION 4**

**4.1** **Subjects 4**

**4.2** **Target Sample Size 4**

**4.3** **Inclusion Criteria 4**

**4.4** **Exclusion Criteria 4**

**4.5** **Enrollment/Allocation Procedures 5**

4.5.1 Enrollment 5

4.5.2 Preparation of Screening Log/Subject Registration List 5

4.5.3 Preparation of the Subject Identification Code List 5

4.5.4. Subject Allocation 6

**5** **STUDY METHOD 6**

**5.1** **Study Design 6**

**5.2** **Dosage and Administration 6**

**5.3** **Study Completion, Subject Discontinuation, and Withdrawal 6**

5.3.1 Study Completion 6

5.3.2 Subject Discontinuation or Withdrawal 6

5.3.3 Discontinuation Procedure 6

5.3.4 Subject Replacement 7

5.3.5 Management of Subjects 7

**6** **CLINICAL STUDY MATERIAL 7**

**6.1** **Proprietary Name and Manufacturer 7**

**6.2** **Constitents, Dosage Form, Storage 7**

**6.3** **Dosage and Administration Method 8**

**6.4** **Supply and Delivery 8**

**6.5** **Storage and Handling 8**

**6.6** **Retrieval 8**

**7** **TESTS, EVALUATIONS, AND TEST PERIOD 8**

**7.1** **Test and Evaluation Items 8**

7.1.1 Screening Items 8

7.1.2 Pre-dose Tests 9

7.1.3 Tests During the Study 9

7.1.4 Tests at the End of the Study 9

7.1.5 Identification of TJ-43 Constituents in Plasma 9

7.1.6 Safety Parameters 10

**7.2** **Schedule of Time and Events 10**

**8** **CRITERIA FOR EVALUATION AND ANALYSIS 13**

**8.1** **Target Population 13**

8.1.1 Target Population for Pharmacokinetic Analysis 13

8.1.2 Target Population for Safety Analysis 13

**8.2** **Endpoints 13**

8.2.1 Pharmacokinetics 13

8.2.2 Safety Assessment 13

8.2.3 Fixation of the Analysis Set 14

**8.3** **Statistical Analysis Protocol 14**

**9** **ADVERSE EVENTS 14**

**9.1** **Definition 14**

9.1.1 Adverse Events 14

9.1.2 Serious Adverse Event 14

9.1.3 Adverse Drug Reaction 14

9.1.4 Causality Assessment 14

9.1.5 Severity of Adverse Events 15

**9.2** **Monitoring Adverse Events 15**

9.2.1 Handling of Adverse Events 15

**9.3** **Submission of New Information 15**

**9.4** **Expected Adverse Drug Reactions 16**

**10** **ETHICAL CONSIDERATIONS 16**

**10.1** **Compliance with Ethical Principles 16**

**10.2** **Institutional Review Board 16**

**10.3** **Informed Consent 16**

10.3.1 When and How to Obtain Informed Consent 16

10.3.2 Elements of Informed Consent 16

10.3.3 Approval and Revision of Informed Consent 17

**10.4** **Confidentiality 17**

**10.5** **Subject Stipend 17**

**10.6** **Health Damage Compensation and Insurance 17**

**10.7** **Study Completion or Premature Termination and Suspension 17**

10.7.1 Study Completion 17

10.7.2 Criteria for Study Termination or Suspension 18

10.7.3 Procedures for Premature Termination or Suspension 18

10.7.4 Procedures for Resuming the Study 18

**11** **PROTOCOL COMPLIANCE AND DEVIATION(S) OR CHANGE(S) AND AMENDMENT(S) 18**

**11.1** **Protocol Compliance 18**

**11.2** **Protocol Deviation(s) or Change(s) 18**

**11.3** **Protocol Amendment(s) 19**

**12** **DATA COLLECTION AND STORAGE 19**

**12.1** **Case Report Form 19**

**12.2** **Storage of Records 19**

**13** **SOURCE DOCUMENT VERIFICATION 19**

**13.1** **Source Document Specifications 19**

**13.2** **Procedure for Direct Access 19**

**13.3** **Evaluation and Handling of Verification of Results 19**

**14** **QUALITY CONTROL AND QUALITY ASSURANCE 20**

**14.1** **Quality Control 20**

**14.2** **Quality Assurance 20**

**15** **PUBLICATION POLICY 20**

**16** **STUDY PERIOD 20**

**17** **STUDY ORGANIZATION 20**

**17.1** **Sponsor 20**

17.1.1 Sponsor 20

17.1.2 Sponsor’s Study Organization 20

17.1.3 Medical Advisor 20

17.2.1 Testing Facility for TJ-43 Constituents in Plasma 21

17.2.2 Testing Facility for Exploratory Retrieval of Metabolites of TJ-43 Constituents in Plasma 21

**17.3** **Testing Facility for TJ-43 Constituents in the Drug Lot 21**

**17.4** **Research Facility and Principal Investigator 21**

**18** **REFERENCES 21**

**19** **STATISTICIAN AND MEDICAL ADVISOR (MEDICAL EXPERT) VERIFICATION 23**

**20** **STATEMENT OF COMPLIANCE WITH THE PROTOCOL 24**

# 1 STUDY SYNOPSIS

## **1.1 TITLE**

Pharmacokinetic study of single administration of rikkunshito (TJ-43) in healthy volunteers

## **1.2 STUDY OBJECTIVE**

To evaluate the pharmacokinetics and safety of Tsumura Rikkunshito Extract Granules (for prescription, hereinafter referred to as “TJ-43”) after single oral doses of 7.5 g (1 sachet 2.5 g × 3 sachets), 5.0 g (1 sachet 2.5 g × 2 sachets), and 2.5 g (1 sachet 2.5 g × 1 sachet) in healthy volunteers

## **1.3 ENDPOINTS**

Pharmacokinetics and safety assessment

## **STUDY POPULATION**

### 1.4.1 Subjects

Healthy volunteers who satisfy all inclusion criteria and do not meet the exclusion criteria

### 1.4.2 Target Sample Size

21 subjects

### 1.4.3 Inclusion Criteria

1) Japanese subjects

2) Subjects aged 20–44 years, inclusive (at the time of informed consent)

3) Subjects with a body mass index (BMI) of 18.5–25 kg/m^2^

4) Both genders

5) Subjects willing and able to provide written and signed informed consent

### 1.4.4 Exclusion Criteria

1) Subjects with a history of allergic reactions to drugs and food(s)

2) Female subjects who are pregnant, breastfeeding, or planning to become pregnant

3) Subjects who have participated in another clinical study within the past 16 weeks

4) Subjects who have had their blood drawn or who have donated >400 mL of blood within 12 weeks of study drug administration

5) Subjects with a history of a significant hepatic, cardiovascular, or hematological disease

6) Subjects who are under treatment for a disease

7) Subjects who are not able to stop smoking or drinking from 3 days prior to study drug administration until completion of each treatment phase of the study

8) Subjects who are not able to stop using medications (e.g., general cold, anti-allergy, anti-fungal, and Kampo medications) from 7 days prior to study drug administration until completion of each treatment phase of the study

Subjects who consumed supplements* containing Atractylodes Lancea Rhizome, Ginseng, Pinellia Tuber, Poria Sclerotium, Jujube, citrus fruits (Citrus Unshiu Peel), Glycyrrhiza, ginger, and vitamin P within 3 days prior to study drug administration

10) Subjects who used other agents from 7 days prior to study drug administration until completion of each treatment phase of the study

11) Subjects with abnormal clinical laboratory values in screening tests or pre-administration tests that were considered inappropriate for the study by the principal investigator/subinvestigator

12) Subjects who are deemed ineligible for other reasons by the principal investigator/subinvestigator

13) Subjects who test positive for HCV antibody, HBs antigen, or HIV antibody

* Supplements are products that contain any of vitamins, minerals, herbs, and amino acids and aim to supplement a regular diet.

## **1.5 STUDY DESIGN**

Randomized crossover study

## **1.6 DOSAGE AND ADMINISTRATION**

Single oral administration of TJ-43 (for prescription) at doses of 7.5 g (1 sachet 2.5 g × 3 sachets), 5.0 g (1 sachet 2.5 g × 2 sachets), and 2.5 g (1 sachet 2.5 g × 1 sachet)

## **1.7 SCHEDULE OF TIME AND EVENTS**

Subjects will be randomly assigned to Groups A–C after enrollment, and 3 different dosages will be administered to the same subject at 3 separate times. The 3 dosage periods will be designated as phase I, phase II, and phase III, with a washout period of greater than 4 weeks between each treatment period, i.e., between phases I and II and between phases II and III (Table 1).

Table 1 Dosing Schedule

|  | Phase I | Washout period | Phase II | Washout period | Phase III |
| --- | --- | --- | --- | --- | --- |
| Group A | 7.5 g | >4 weeks | 2.5 g | >4 weeks | 5.0 g |
| Group B | 2.5 g | >4 weeks | 5.0 g | >4 weeks | 7.5 g |
| Group C | 5.0 g | >4 weeks | 7.5 g | >4 weeks | - 1. g |

## **1.8 OTHER FACTORS FOR CONTROL**

1) Foods and drinks containing caffeine, alcohol, citrus fruits (grapefruit, orange, tangerine, etc.), vitamin P, ginseng, ginger, Glycyrrhiza (listed on a food label), and garlic (including spices) (see attached references) are prohibited from 3 days prior to study drug administration until completion of each treatment phase of the study. Supplements containing constituents of rikkunshito (Atractylodes Lancea Rhizome, Ginseng, Pinellia Tuber, Poria Sclerotium, Jujube, Citrus Unshiu Peel, Glycyrrhiza, Ginger), processed ginger, and vitamin P are also prohibited. However, foods and drinks that do not contain the above (see attached references) may be consumed at any time. Subjects must have fasted from 20:00 on the eve of study drug administration until 4 h post-dose.

2) Smoking: Smoking is prohibited from 3 days prior to study drug administration until completion of each treatment phase of the study.

3) Use of other medications: Use of other medications is prohibited from 7 days prior to study drug administration until completion of each treatment phase of the study.

## **1.9 STUDY PERIOD**

September 2013–May 2014

## **1.10 TESTING INSTITUTION**

Kochi Medical School Hospital

Kohasu Oko-cho, Nankoku-shi, Kochi 783-8505, Japan

TEL: +81-88-866-5811

## **1.11 SPONSOR**

Tsumura & Co., 2-17-11 Akasaka, Minato-ku, Tokyo 107-8521, Japan

Contact information: Kampo Research Planning Department

TEL: +813-6361-7184, FAX: +813-5574-6664

# 2 BACKGROUND

Rikkunshito, a traditional medicine described in the classic literature of Kampo [Manbyokaishun], is composed of 8 crude drugs: Atractylodes Lancea Rhizome, Ginseng, Pinellia Tuber, Poria Sclerotium, Jujube, Citrus Unshiu Peel, Glycyrrhiza, Ginger. Rikkunshito is a combination of shikunshito (Atractylodes Lancea Rhizome or Atractylodes Rhizome, Poria Sclerotium, Ginseng, Jujube, Ginger, and Glycyrrhiza) and nichinto (Pinellia Tuber, Citrus Unshiu Peel, Poria Sclerotium, Ginger, and Glycyrrhiza).

Rikkunshito has been widely used for the treatment of “poor digestion, poor appetite, dull feeling in the stomach, fatigue, and cold feet and hands due to anemia.” TJ-43 is a convenient granule form of rikkunshito, which is manufactured by Tsumura with its proprietary dry granulation method and officially approved as a Kampo extract formulation for prescription under the provisions stipulated in “Report No. 120 of the Central Pharmaceutical Affairs Council, Ministry of Health and Welfare, dated May 31, 1985.” TJ-43 has broad clinical applications for the treatment of various digestive symptoms, including gastritis, stomach ache, dull feeling in the stomach, abdominal fullness, loss of appetite, and heartburn^1)-3)^. In recent years, it has been reported that TJ-43 is effective for improving digestive symptoms in patients with gastroesophageal reflux disease^4)-5)^ and functional dyspepsia^2),6)^. Evidence suggests that TJ-43 improves the above-mentioned digestive conditions through functions such as gastric adaptive relaxation^6),7)^, promotion of gastric emptying^8)-10)^, improvement of esophagus clearance^5)^, and regulation of ghrelin secretion and response^11)-15)^. Furthermore, several studies have focused on the active ingredients of TJ-43. It has been reported that atractylodin in Atractylodes Lancea Rhizome promotes ghrelin receptor activity^15)^; pachymic acid in Poria Sclerotium suppresses metabolism of the active form of ghrelin^16)^; and heptamethoxyflavone, nobiletin, and naringenin in Citrus Unshiu Peel as well as liquiritigenin and isoliquiritigenin in Glycyrrhiza promote ghrelin secretion^11)^. While there are several reports on the pharmacological effects of TJ-43, pharmacokinetic studies of the Kampo formulation TJ-43 are limited because of the difficulty in conducting such studies on account of the multiple constituents involved. Although pharmacokinetic studies of isolated constituents such as Glycyrrhiza are occasionally found in literature^17),18)^, no appropriate pharmacokinetic profiling of orally administered constituent crude drug has been conducted clinically.

Therefore, as a prelude to the clinical pharmacokinetic study of TJ-43, we performed a study “Exploratory Pharmacokinetic Study of Rikkunshito (TJ-43) in Healthy Volunteers” to identify the constituents absorbed after administration of TJ-43 in humans in April 2012^19)^. As a result, presence of the active constituents of TJ-43, such as atractylodin, a constituent of Atractylodes Lancea Rhizome; pachymic acid, a constituent of Poria Sclerotium; and heptamethoxyflavone, a constituent of Citrus Unshiu Peel, was confirmed in plasma.

Considering the background and results described above, a quantitative clinical pharmacokinetic study on atractylodin, pachymic acid, heptamethoxyflavone, liquiritigenin, isoliquiritigenin, nobiletin, and naringenin was planned to elucidate the pharmacokinetics of each constituent after administration of TJ-43. Long-term use of large amounts of Glycyrrhiza, an ingredient of TJ-43, may cause pseudoaldosteronism. The causal constituents are believed to be glycyrrhetinic acid and glycyrrhetinic acid monoglucuronide, which are metabolites of glycyrrhizin, a constituent of Glycyrrhiza^18)^. To confirm the presence of the constituents of Glycyrrhiza, glycyrrhetinic acid was also analyzed. A quantitative clinical pharmacokinetic study of yokukansan, which contains Glycyrrhiza as an ingredient, showed extremely low levels of glycyrrhetinic acid monoglucuronide^20)^. Therefore, glycyrrhetinic acid monoglucuronide was excluded from analysis in this study. The findings of this study are expected to provide important data regarding adverse drug reactions (ADRs).

We believe that a quantitative pharmacokinetic study of TJ-43 in humans would be extremely useful for understanding the relationship between the basic pharmacological action and constituents of TJ-43. Furthermore, it is important to predict the possibility of accumulation of unchanged compounds or metabolites by evaluating drug clearance.

The present study will be conducted in accordance with the trial protocol and standards specified under the Ministry of Health, Labour and Welfare (MHLW) ordinance regarding Good Clinical Practice (GCP), MHLW ordinance regarding Good Post-Marketing Surveillance/Study Practice (GPSP), and Article 14, Paragraph 3, and Article 80, Paragraph 2, of the Pharmaceutical Affairs Law.

# 3 STUDY OBJECTIVE

## **3.1 STUDY OBJECTIVE**

To evaluate the pharmacokinetics and safety of TJ-43 after single oral doses of 7.5 g (1 sachet 2.5 g × 3 sachets), 5.0 g (1 sachet 2.5 g × 2 sachets), and 2.5 g (1 sachet 2.5 g × 1 sachet) in healthy volunteers.

**3.2 TYPE OF STUDY**

Quantitative study (post-marketing clinical study)

# 4 STUDY POPULATION

**4.1** **SUBJECTS**

# Healthy volunteers who satisfy all inclusion criteria and do not meet the exclusion criteria. A healthy volunteer is a person who does not have a chronic disease and who is able to lead his/her daily life without difficulty.

**4.2 TARGET SAMPLE SIZE**

21 completed subjects

[Rationale for Sample Size Determination]

The conventional sample size required to evaluate pharmacokinetics is 15 subjects. The sample size for this study was set to 21 subjects considering withdrawal, etc. For the definition of the term “completed,” refer to 5.3.1.

**4.3 INCLUSION CRITERIA**

1) Japanese subjects

2) Subjects aged 20–44 years, inclusive (at the time of informed consent)

3) Subjects with a BMI of 18.5–25 kg/m^2^

4) Both genders

5) Subjects willing and able to provide written and signed informed consent

[Rationale for Inclusion Criteria]

The subjects of this study are Japanese. The purpose of setting the lower age limit is to select adults with the ability to make an informed decision about study participation, whereas the upper age limit serves to dampen the physiological effects of aging from older subjects. BMI within the normal range as specified by the Japan Society for the Study of Obesity is included to minimize the effects of obesity on the distribution of the study drug.

**4.4 EXCLUSION CRITERIA**

1) Subjects with a history of allergic reactions to drugs and food(s)

2) Female subjects who are pregnant, breastfeeding, or planning to become pregnant

3) Subjects who have participated in another clinical study within the past 16 weeks

4) Subjects who have had their blood drawn or who have donated >400 mL of blood within 12 weeks of study drug administration

5) Subjects with a history of a significant hepatic, cardiovascular, or hematological disease

6) Subjects who are under treatment for a disease

7) Subjects who are not able to stop smoking or drinking from 3 days prior to study drug administration until completion of each treatment phase of the study

8) Subjects who are not able to stop taking medications (e.g., general cold, anti-allergy, anti-fungal, and Kampo medications) from 7 days prior to study drug administration until completion of each treatment phase of the study

9) Subjects who consumed supplements* containing Atractylodes Lancea Rhizome, Ginseng, Pinellia Tuber, Poria Sclerotium, Jujube, citrus fruits (Citrus Unshiu Peel), Glycyrrhiza, ginger, and vitamin P within 3 days prior to study drug administration

10) Subjects who used other agents from 7 days prior to study drug administration until completion of each treatment phase of the study

11) Subjects with abnormal clinical laboratory values in screening tests or pre-administration tests that were considered inappropriate for the study by the principal investigator/subinvestigator

12) Subjects who are deemed ineligible for other reasons by the principal investigator/subinvestigator

13) Subjects who test positive for HCV antibody, HBs antigen, or HIV antibody

* Supplements are products that contain any of vitamins, minerals, herbs, and amino acids and aim to supplement a regular diet.

[Rationale for Exclusion Criteria]

1)–5) ensure the safety of the subjects, 6)–13) eliminate subjects who may interfere with the objectives of the study, and 13) ensures the safety of the personnel in charge of collecting and analyzing blood from subjects.

**4.5 ENROLLMENT/ALLOCATION PROCEDURES**

### 4.5.1 Enrollment

The principal investigator/subinvestigator will select and enroll subjects according to the following procedures.

1) The details of the study will be explained to the prospective participants who meet all of the inclusion criteria described in 1)–4) and do not meet any of the exclusion criteria described in 1)–13) and who provide written informed consent to participate in the study.

2) Subject candidates will be selected according to the results of the screening tests.

3) Subjects will be selected from subject candidates according to the results of the pre-administration tests.

### 4.5.2 Preparation of Screening Log/Subject Registration List

Information regarding the date of the consent process (date when the request for an informed consent and information provision was made), existence of consent, and date of the signed consent form for all prospective participants (who have requested and received the informed consent explanations) will be documented by the principle investigator in the “Screening Log/Subject Registration List (Form 1)” regardless of their consent.

Subject identification codes and gender are recorded only for those subjects who have been determined as eligible subjects and who have consented to participation.

### 4.5.3 Preparation of the Subject Identification Code List

Subject identification codes must be assigned to each subject by the principal investigator before study drug administration (subject allocation).

Furthermore, the “Subject Identification Code List (Form 2)” must be prepared separately from the “Screening Log/Subject Registration List” described above. This form must contain the subject identification code, medical record number, and subject name.

The principal investigator must use subject identification codes to identify subjects when reporting adverse events (AEs) and other study-related information.

| Fixed code - Subject number | Subject identification code (sample) |
| --- | --- |
| BR01 - 01, 02, 03, 04 | BR01-01 |

### 4.5.4 Subject Allocation

Subjects who are selected by screening and pre-dose testing will be randomly assigned (allocation ratio of 1:1:1) into 3 groups (Groups A–C) after enrolling with the central allocation system. At this time, the below-mentioned information will also be recorded in the “Subject Identification Code List (Form 2).”

(a) Eligibility

(b) Order of allocation

(c) Allocated group [Group A, B, or C]

# 5 STUDY METHOD

**5.1 STUDY DESIGN**

This is a randomized crossover study. The study drug will be administered to the same subjects after a washout period of greater than 4 weeks.

**5.2 DOSAGE AND ADMINISTRATION**

Subjects will be randomly assigned to Groups A–C after enrollment, and 3 different dosages will be administered to the same subject at 3 separate times. The 3 dosage periods will be designated as phase I, phase II, and phase III, with a washout period of greater than 4 weeks between phases I and II and between phases II and III (Table 1).

Table 1 Dosing Schedule

|  | Phase I | Washout period | Phase II | Washout period | Phase III |
| --- | --- | --- | --- | --- | --- |
| Group A | 7.5 g | >4 weeks | 2.5 g | >4 weeks | 5.0 g |
| Group B | 2.5 g | >4 weeks | 5.0 g | >4 weeks | 7.5 g |
| Group C | 5.0 g | >4 weeks | 7.5 g | >4 weeks | 2.5 g |

**5.3 STUDY COMPLETION, SUBJECT DISCONTINUATION, AND WITHDRAWAL**

### 5.3.1 Study Completion

Each treatment phase will be considered complete after blood sampling and testing at the end of 48 h after study drug administration.

The study will conclude after completion of the 3 treatment phases.

### 5.3.2 Subject Discontinuation or Withdrawal

The investigator may terminate or withdraw a subject from the study in case of the following:

1) If the subject requests withdrawal or declines participation

2) If the subject experiences an AE that undermines continuation of the study in the opinion of the principal investigator

3) If the subject is unable to take the study drug

4) If the subject is judged as ineligible to continue the study by the principal investigator

### 5.3.3 Discontinuation Procedure

The principal investigator/subinvestigator must document the reason(s) for discontinuation, measures taken, and follow-up in the Case Report Form (CRF). If discontinuation is for safety reasons, appropriate measures must be taken and the subjects should be followed-up to resolution until their safety is assured, except in cases where consent cannot be obtained from the subjects.

### 5.3.4 Subject Replacement

Subjects will be replaced if discontinuation or withdrawal results in a sample size that falls below the required size for the study (15 subjects). The number of withdrawn subjects will be replaced, and the subjects will undergo test procedures beginning from phase I according to their allocated group. Samples collected from participants until discontinuation may be used for analysis as long as consent is obtained from the withdrawn subjects.

### 5.3.5 Management of Subjects

All subjects are under close supervision of the principal investigator/subinvestigator during their participation in the clinical study.

The principal investigator/subinvestigator will instruct all subjects to comply with the following directions.

1) Foods and drinks

Foods and drinks containing caffeine, alcohol, citrus fruits (grapefruit, orange, tangerine, etc.), vitamin P, Ginseng, Ginger, Glycyrrhiza (listed on a food label) and garlic (including spices) (see attached reference) are prohibited from 3 days prior to study drug administration until completion of each treatment phase of the study. Supplements containing constituents of rikkunshito (Atractylodes Lancea Rhizome, Ginseng, Pinellia Tuber, Poria Sclerotium, Jujube, Citrus Unshiu Peel, Glycyrrhiza,

Ginger), Processed Ginger, and vitamin P are also prohibited. However, foods and drinks that do not contain the above (see attached references) may be consumed at any time. Subjects must have fasted from 20:00 on the eve of study drug administration to 4 h post-dose.

2) Smoking

Smoking is prohibited from 3 days prior to study drug administration until completion of each treatment phase of the study.

3) Use of other drugs

Use of other drugs is prohibited from 7 days prior to study drug administration until completion of each treatment phase of the study.

# 6 CLINICAL STUDY MATERIAL

# 6.1 PROPRIETARY NAME AND MANUFACTURER

Study drug: Tsumura Rikkunshito Extract Granule (for prescription)

Code number: TJ-43

Manufacturer: Tsumura & Co.

## **6.2 CONSTITUENTS, DOSAGE FORM, STORAGE**

Constituents/Content: 7.5 g of TJ-43 contains 4.0 g of dried extract of the following ratio of crude drugs.

JP Atractylodes Lancea Rhizome 4.0 g JP Jujube 2.0 g

JP Ginseng 4.0 g JP Citrus Unshiu Peel 2.0 g

JP Pinellia Tuber 4.0 g JP Glycyrrhiza 1.0 g

JP Poria Sclerotium 4.0 g JP Ginger 0.5 g

(JP: The Japanese Pharmacopoeia)

Additives: JP Magnesium Stearate, JP Lactose Hydrate, Sucrose Esters of Fatty Acids

Dosage form: Granules

Packaging: One heat-sealed sachet contains 2.5 g of extract granules

Storage: Store in a cool, dry place. Avoid contact with moisture and direct sunlight.

**6.3 DOSAGE AND ADMINISTRATION METHOD**

Single oral administration of TJ-43 at doses of 7.5 g (1 sachet 2.5 g × 3 sachets), 5.0 g (1 sachet 2.5 g × 2 sachets), and 2.5 g (1 sachet 2.5 g × 1 sachet)

[Rationale for Dosage]

In this study, the doses of 7.5 g, 5.0 g, and 2.5 g (at a constant difference of 2.5) have been selected on the basis of the following 3 conditions.

A total of 7.5 g is usually administered over 2 or 3 doses daily.

1. Determination of the maximum dose of 7.5g that is well tolerated in subjects as a single dose

AEs associated with TJ-43 did not occur after single oral administration of 7.5 g TJ-43 in a previously conducted preliminary pharmacokinetics study^19)^.

1. Determination of the minimum dose of 2.5 g based on TJ-43 dosage and administration

The minimum dose of TJ-43 has been set at 2.5 g, which is the average prescribed single dose.

1. Measurability of concentrations

Based on the results of the preliminary pharmacokinetics study^19)^, measurement of TJ-43 constituents in plasma is probable with the minimal administration dosage of 2.5 g due to the improved and sensitive analytical method.

The maximum plasma concentration of each constituent in the preliminary pharmacokinetics study^19)^ (7.5 g dosage) was 0.228 ± 0.116 ng/mL for pachymic acid, 0.0475 ± 0.0297 ng/mL for isoliquiritigenin, 1.84 ± 0.626 ng/mL for atractylodin, 0.0626 ± 0.0436 ng/mL for nobiletin, 0.253 ± 0.301 ng/mL for heptamethoxyflavone, 0.162 ± 0.0392 ng/mL for naringenin, and 64.7 ± 11.8 ng/mL for glycyrrhetinic acid. The lower limits of quantitation for the constituents are 0.1 ng/mL for pachymic acid, 0.02 ng/mL for isoliquiritigenin, 1 ng/mL for atractylodin, 0.02 ng/mL for nobiletin, 0.02 ng/mL for heptamethoxyflavone, 0.1 ng/mL for naringenin, and 0.1 ng/mL for glycyrrhetinic acid.

**6.4 SUPPLY AND DELIVERY**

The clinical study material will be supplied and delivered by the sponsor to the institution for free after the study contract is signed between the two parties.

**6.5 STORAGE AND HANDLING**

The study drug administrator will store and handle the clinical study material in an appropriate manner.

**6.6 RETRIEVAL**

The study drug administrator will retrieve all unused clinical study material after inspection by the sponsor.

**7 TESTS, EVALUATIONS, AND TEST PERIOD**

**7.1 TEST AND EVALUATION ITEMS**

**7.1.1 Screening Items**

The principal investigator/subinvestigator will conduct the following medical interview, clinical observations, and laboratory tests 1 week prior to the start of the trial and select prospective subjects on the basis of the test results. The screening date’s margin of error is ±1 day.

Demographics: date of birth, gender, race, present illness, current medications, history of drug and food allergies, past medical history, alcohol intake, smoking history

Clinical findings: subjective symptoms, objective findings, edema

Physical examination: height, weight, BMI, body temperature (axillary), blood pressure (seated), pulse (seated)

Laboratory tests: 1) Hematology

erythrocytes, leukocytes, platelets, hemoglobin, hematocrit

2) Blood chemistry

total protein, BUN, creatinine, uric acid, AST, ALT, T-Bil, ALP, γ-GTP, Alb, PT, TC, CRP, HbA1c, K

3) Urinalysis

urine sugar

Urine pregnancy test (female)

Viral tests: HCV antibody, HBs antigen, HIV antibody

**7.1.2 Pre-dose Tests**

The principal investigator/subinvestigator will perform the following clinical observations and laboratory tests 1 day prior to study drug administration and select prospective subjects on the basis of comprehensive analysis of the test results.

Clinical findings: subjective symptoms, objective findings, edema

Physical examination: body temperature (axillary), blood pressure (seated), pulse (seated)

Laboratory tests: 1) Hematology

erythrocytes, leukocytes, platelets, hemoglobin, hematocrit

2) Blood chemistry

total protein, BUN, creatinine, uric acid, AST, ALT, T-Bil, ALP, γ-GTP, Alb, PT, TC, CRP, K

Urine pregnancy test (female): At the time of screening in phase I

**7.1.3 Tests During the Study**

Clinical findings: subjective symptoms, objective findings

**7.1.4 Tests at the End of the Study**

At the end of each treatment phase, the principal investigator/subinvestigator will perform the following clinical observations and laboratory tests.

Clinical findings: subjective symptoms, objective findings, edema

Physical examination: body temperature (axillary), blood pressure (seated), pulse (seated)

Laboratory tests: 1) Hematology

erythrocytes, leukocytes, platelets, hemoglobin, hematocrit

2) Blood chemistry

total protein, BUN, creatinine, uric acid, AST, ALT, T-Bil, ALP, γ-GTP, Alb, PT, TC, CRP, K

**7.1.5 Identification of TJ-43 Constituents in Plasma**

Total blood drawn in this study is 524 mL {screening tests: 8 mL × 1, 172 mL for each of the 3 phases [(clinical tests: 8 mL × 2, pharmacokinetics; 12 mL × 13) × 3]}

1) Blood collection (pharmacokinetics): Sampling time

Pre-dose sample and post-dose samples at 15 min, 30 min, 1 h, 2 h, 3 h, 4 h, 6 h, 8 h, 10 h, 12 h, 24 h, and 48 h will be collected (13 total samples).

2) Method and handling of sampling

A total of 12 mL of venous blood (for the pharmacokinetic study) will be collected at one time from the subject’s antecubital vein during the sampling time, dispensed into a tube containing heparin sodium as the anticoagulant, and centrifuged at 1700 × *g* for 15 min at 4°C to obtain a plasma sample. The obtained plasma will be dispensed into tubes as needed and cryopreserved below −20°C until analysis.

3) Acceptable range and handling of sampling time error

Sampling times will be recorded at the start of collection. The acceptable sampling time windows for post-dose collection at 15 min, 30 min, and 1 h will be set within 10% of the sampling times (15 min ± 1.5 min, 30 min ± 3 min, and 1 h ± 6 min, respectively). The acceptable sampling time windows for 2-h, 3-h, and 4-h collection will be uniformly set within ±10 min (2 h ± 10 min, 3 h ± 10 min, and 4 h ± 10 min, respectively). The acceptable sampling time windows for 6-h, 8-h, 10-h, 12-h, 24-h, and 48-h collection will be uniformly set within ±30 min (6 h ± 30 min, 8 h ± 30 min, 10 h ± 30 min, 12 h ± 30 min, 24 h ± 30 min, and 48 h ± 30 min, respectively). Deviations from sampling time windows will be indicated in the CRF. Deviated samples may also be submitted for analysis. The analyzed data should be handled according to the pharmacokinetic analysis plan, which will be prepared separately.

[Rationale for Sampling Time Windows]

It was planned to collect 13 samples (12 mL each) in this study starting with pre-dose (used as a blank plasma) and at 15 min, 30 min, 1 h, 2 h, 3 h, 4 h, 8 h, 6 h, 10 h, 12 h, 24 h, and 48 h post-dose.

Based on the results of the preliminary pharmacokinetic study and existing reports^19)^, the time to peak maximum concentration in plasma (*t*_max_) for most of the 8 constituents selected for quantitative analysis is within 2 h. However, it has also been confirmed that the *t*_max_ values of naringenin and glycyrrhetinic acid are approximately between 6 and 8 h, which is relatively longer compared with the values of the other constituents. Therefore, the sampling time was determined as follows.

Considering the predicted *t*_max_ of each of the 8 constituents, the sampling time that enables evaluation of the pharmacokinetic characteristics of each constituent was determined. Furthermore, the half-life of glycyrrhetinic acid, which has the longest half-life among the 8 constituents in this trial, is approximately 10 h; therefore, a final sampling time of 48 h after administration of the study drug was chosen so that evaluation up to 3–5 times the half-life was possible. According to the guidelines for pharmacokinetic studies in Japan, a measurement period of up to 3 times the half-life of a measured material is required, whereas sampling up to 5 times the half-life is required by the FDA. In this study, the 48-h sampling time window was planned to fulfill both guidelines.

The volume of blood to be collected was determined by considering the amount required for analysis (approximately 5 mL was calculated for each pharmacokinetic analysis, including the amount for possible reanalysis) and was decided to be 12 mL (using heparin sodium as the anticoagulant).

### 7.1.6 Safety Parameters

Clinical findings, physical examination findings, laboratory examination findings

**7.2 SCHEDULE OF TIME AND EVENTS**

Each laboratory test and clinical observation will be performed according to the study schedule in Table 2

Table 2 Study schedule

|  | Day-7 | Phase I | | >4  weeks | Phase II | | >4  weeks | Phase III | |
| --- | --- | --- | --- | --- | --- | --- | --- | --- | --- |
|  |  | 1-Day-1 | 1-Day0~1-Day+2 | Washout Period | 2-Day-1 | 2-Day0 ~  2-Day+2 | Washout Period | 3-Day-1 | 3-Day0~  3-Day+2 |
|  | Screening | Pre-dose | Dosing and  blood sampling  (until 48 h) |  | Pre-dose | Dosing and blood sampling (until 48 h) |  | Pre-dose | Dosing and blood sampling  (until 48 h) |
| Informed consent | 〇 |  |  |  |  |  |  |  |  |
| Enrollment |  | 〇 |  |  |  |  |  |  |  |
| Allocation |  | 〇 |  |  |  |  |  |  |  |
| Demographics | 〇 |  |  |  |  |  |  |  |  |
| Height, weight, BMI | 〇 |  |  |  |  |  |  |  |  |
| Blood pressure, pulse, body temperature | 〇 | 〇 | 〇 |  | 〇 | 〇 |  | 〇 | 〇 |
| Clinical examination | 〇 | 〇 | 〇 |  | 〇 | 〇 |  | 〇 | 〇 |
| Urine pregnancy test | 〇 |  |  |  | 〇 |  |  | 〇 |  |
| Lab tests | 〇 | 〇 | 〇 |  | 〇 | 〇 |  | 〇 | 〇 |
| TJ-43 administration  Group A  Group B  Group C |  |  | 〇  7.5 g  2.5 g  5 g |  |  | 〇  2.5 g  5 g  7.5 g |  |  | 〇  5 g  7.5 g  2.5 g |
| Pharmacokinetics |  |  | 〇 |  |  | 〇 |  |  | 〇 |
| Safety  assessment |  |  |  |  |  |  |  |  |  |

Each laboratory test and clinical observation will be performed according to the study schedule in Table 3 (sample time schedule is shown).

Table 3 Study Schedule 2 (each phase)^Note1^

|  | Day  -7 | Day  -1 | Day 0 | | | | | | | | | | | | Day  +1 | Day  +2 |
| --- | --- | --- | --- | --- | --- | --- | --- | --- | --- | --- | --- | --- | --- | --- | --- | --- |
|  | Screening | Pre-dosing | Immediately before dosing | 0  h | 15  min | 30  min | 1  h | 2  h | 3  h | 4  h | 6  h | 8  h | 10  h | 12  h | 24  h | 48  h |
|  |  |  | 7:  30 | 8:  00 | 8:  15 | 8:  30 | 9:  00 | 10:00 | 11:00 | 12:00 | 14:00 | 16:00 | 18:00 | 20:00 | 8:  00 | 8:  00 |
| Informed consent | ○ |  |  |  |  |  |  |  |  |  |  |  |  |  |  |  |
| Subject roster |  | ○ |  |  |  |  |  |  |  |  |  |  |  |  |  |  |
| Allocation |  | ○ |  |  |  |  |  |  |  |  |  |  |  |  |  |  |
| Demographics | ○ |  |  |  |  |  |  |  |  |  |  |  |  |  |  |  |
| Height, weight,  BMI | ○ |  |  |  |  |  |  |  |  |  |  |  |  |  |  |  |
| Blood pressure, pulse,  body temperature | ○ | ○ |  |  |  |  |  |  |  |  |  |  |  |  |  | ○ |
| Clinical examination | ○ | ○ |  |  |  |  |  |  |  |  |  |  |  |  |  | ○ |
| Urine pregnancy test^Note2^ | ○ | |  |  |  |  |  |  |  |  |  |  |  |  |  |  |
| Lab tests^Note3^  sample volume | ○  8 mL | ○  8 mL |  |  |  |  |  |  |  |  |  |  |  |  |  | ○  8 mL |
| TJ-43 dosing |  |  |  | ○ |  |  |  |  |  |  |  |  |  |  |  |  |
| Meal^Note4^ |  | ○ |  |  |  |  |  |  |  | ○ |  |  | ○ |  | ○ | ○ |
| PK^Note3^  sample volume |  |  | ○  12 mL |  | ○  12 mL | ○  12 mL | ○  12 mL | ○  12 mL | ○  12 mL | ○  12 mL | ○  12 mL | ○  12 mL | ○  12 mL | ○  12 mL | ○  12 mL | ○  12 mL |
| Safety assessment |  |  |  |  |  |  |  |  |  |  |  |  |  |  |  |  |

Note 1: The schedule is for phase I. Screening tests conducted 7 days prior to administration of TJ-43 will not be performed in phases II and III, whereas pre-dose tests conducted 1 day prior to administration and other tests scheduled thereafter will be performed.

Note 2: This will be conducted in females only. It will be performed at the time of screening in phase I. It will be conducted 1 day prior to administration of J-43 in phases II and III.

Note 3: The total blood drawn will be 524 mL {screening: 8 mL × 1, 172 mL for each of the 3 phases [(8 mL × 2 for laboratory tests, 12 mL × 13 for pharmacokinetic) × 3]}. In addition, general urinalysis (urine sugar) will be performed at the time of screening only.

Note 4: Foods and drinks (including spices) (see attached reference) containing caffeine, alcohol, citrus fruits (grapefruit, orange, tangerine, etc.), vitamin P, ginseng, ginger, kanzo (listed on a food label), and garlic are prohibited from 3 days prior to study drug administration until completion of each treatment phase of the study. Supplements containing constituents of rikkunshito (Atractylodes Lancea Rhizome, Ginseng, Pinellia Tuber, Poria Sclerotium, Jujube, Citrus Unshiu Peel, Glycyrrhiza, and Ginger), Processed Ginger, and vitamin P are also prohibited. However foods and drinks that do not contain the above (see attached references) may be consumed at any time. Subjects must have fasted from 20:00 on the eve of study drug administration to 4 h post-dose.

**8. CRITERIA FOR EVALUATION AND ANALYSIS**

**8.1 TARGET POPULATION**

**8.1.1 Target Population for Pharmacokinetic Analysis**

Registered subjects who satisfy the inclusion criteria and do not meet the exclusion criteria as well as those who have completed sampling for pharmacokinetic analysis after administration of the study drug

**8.1.2 Target Population for Safety Analysis**

Subjects who have taken the study drug

**8.2 ENDPOINTS**

**8.2.1 Pharmacokinetics**

Pharmacokinetics will be evaluated using the collected blood samples by measuring plasma concentration of 8 constituents (atractylodin, pachymic acid, heptamethoxyflavone, liquiritigenin, isoliquiritigenin, nobiletin, naringenin, and glycyrrhetinic acid) and calculating pharmacokinetic parameters such as maximum drug concentration in plasma (*C*_max_), maximum drug concentration time in plasma (*t*_max_), and area under the blood concentration–time curve (AUC) from plasma concentration of the 8 constituents for which validated analytical methods have been established.

The detailed method of plasma concentration analysis of the 8 constituents will be written in the study plan, which will be prepared separately. Shin Nippon Biomedical Laboratories and Mitsubishi Medical Medience will perform the analyses; however, additional analyses, including preliminary metabolite screening, will be conducted as required.

The details of pharmacokinetic analyses will be documented in the pharmacokinetic analysis plan, which will be prepared separately. Tsumura Laboratories will perform pharmacokinetic analysis by following this document. Data from blood collected outside the sampling windows will be handled as described in the pharmacokinetic analysis plan.

Various pharmacokinetic data, including plasma concentration measurements, may be used for separate analysis at a later time.

The concentration of the 8 constituents in TJ-43 drug lots used for pharmacokinetic analysis will be obtained when plasma concentration analysis of the TJ-43 lot is performed by Sumika Chemical Analysis Service, LTD.

**8.2.2 Safety Assessment**

Safety will be comprehensively evaluated from the overall assessment of clinical findings, physical examination, and laboratory tests performed prior to and during the study. The results of these tests performed before administration of the study drug will become the baseline values and changes observed after dosing will be compared with these baseline values.

[Abnormal changes]

-The principal investigator will compare the values obtained before dosing with those obtained after dosing; if the change between the two values is more than 20%, it will be considered as an abnormal change.

-Abnormal changes will be handled as AEs and recorded in the AE list.

**8.2.3 Fixation of the Analysis Set**

The sponsor will fix the target population for pharmacokinetic-related analysis and safety-related analysis according to Tsumura-GCP/SOP/11.

**8.3 STATISTICAL ANALYSIS PLAN**

Methods for identifying and classifying the endpoints for analysis, as well as the timeframe and methods for conducting analyses, should follow the “Statistical Analysis Plan (Appendix 2).” Furthermore, statistical analysis will be performed when the guideline for statistical analysis is prepared. Pharmacokinetics will be analyzed according to the Pharmacokinetic Analysis Plan, which will be prepared separately from the “Statistical Analysis Plan (Appendix 2).”

**9 ADVERSE EVENTS**

**9.1 DEFINITION**

**9.1.1 Adverse Events**

An AE is any unfavorable or unintended medical sign (including abnormal laboratory values), symptom, or disease in a subject following exposure to the study drug, regardless of a causal relationship with the study drug. A condition that develops during the study is also included.

**9.1.2 Serious Adverse Event**

A serious adverse event (SAE) is defined as an AE that results in any of the following outcomes:

(1) Death

(2) A life-threatening condition

(3) Hospitalization or prolongation of existing hospitalization

(4) Persistent or significant disability/incapacity

(5) A congenital anomaly or birth defect

(6) Another medically significant event

**9.1.3 Adverse Drug Reaction**

An ADR is defined as an AE for which a causal relationship with the study drug cannot be ruled out (refer to sections 1)–3) of 9.1.4 Causality Assessment).

**9.1.4 Causality Assessment**

The following criteria will be used to characterize causality into 4 classes:

[Causality Classification]

1) Definitely Related

There is evidence to suggest a plausible temporal relationship (including follow-up after termination of treatment) between the study drug and AE. Sufficient evidence to support this relationship is also found.

2) Possibly Related

There is evidence to suggest a reasonable temporal relationship (including follow-up after termination of treatment) between the study drug and AE. The AE is unlikely to be attributed to factors other than the study drug.

3) Possibly Unrelated

There is evidence to suggest a reasonable temporal relationship (including follow-up after termination of treatment) between the study drug and AE. However, factors such as underlying disease, concurrent illness, and concurrent medication/intervention may also be implicated.

4) Not Related

A temporal relationship between the study drug and AE is improbable because other factors such as underlying disease, concurrent illness, and concurrent medication/intervention provide plausible explanations; thus, there is enough evidence to exclude the involvement of the study drug.

**9.1.5 Severity of Adverse Events**

1) Mild:

The event is generally temporary; it does not impair the person’s ability to conduct normal life functions (i.e., normal activities are possible) and does not require treatment.

2) Moderate

The event interferes with the person’s ability to conduct normal life functions (i.e., normal activities are accompanied by discomfort) and requires treatment.

3) Severe

The event severely impairs the person’s ability to conduct normal life functions (i.e., normal activities are onerous) and requires treatment.

**9.2 MONITORING ADVERSE EVENTS**

Any AE must be investigated through inquiry and observation, and if confirmed, the event must be followed and details regarding the following items must be documented in the CRF.

i) type of AE

ii) date of onset

iii) seriousness (1. non-serious, 2. serious)

iv) rationale for seriousness and other medically significant events

v) severity (1. mild, 2. moderate, 3. severe)

vi) treatment (description of intervention if implemented)

vii) outcome [1. recovery (resolution), 2. remission, 3. irresolution (unchanged), 4. complication, 5. unknown]

viii) date of verified outcome

ix) causality (1. definitely related, 2. possibly related, 3. possibly unrelated, 4. not related)

The principal investigator/subinvestigator must document clinically significant, abnormal changes in laboratory test values and their explanations in the CRF.

Causality assessment between the AE and study drug must be documented in the CRF.

**9.2.1 Handling of Adverse Events**

Regardless of causality, the principal investigator must promptly (within 24 h) report any AE considered to be serious (according to the criteria in section 9.1.2) to the affiliated institution head/institutional review board (IRB) and sponsor in person, by telephone, or by fax, take appropriate actions, and also submit a written “Serious Adverse Event Report Form (Form 3)” within few days of the event. In addition, the principal investigator/subinvestigator must provide additional information to the sponsor, institution head, and IRB upon request.

The institution head must seek IRB’s assessment of the institution’s competence to continue the study and take the necessary steps.

The sponsor must immediately report expected serious ADRs according to the package insert and unexpected serious or non-serious ADRs in suspected subjects to the appropriate regulatory agencies in accordance with Tsumura-GCP/SOP and Tsumura-GPSP/SOP, as well as Article 77, Paragraph 4, Item 2, and Article 253 of the Pharmaceutical Affairs Act.

**9.3 SUBMISSION OF NEW INFORMATION**

Upon receipt of new information regarding product document revisions, safety of the study drug, etc., the sponsor must promptly notify the institution head/IRB and the principal investigator in writing and take the necessary measures.

**9.4 EXPECTED ADVERSE DRUG REACTIONS**

Refer to the product document “Tsumura Rikkunshito Extract Granule (Prescription) Package Insert.”

**10. ETHICAL CONSIDERATIONS**

**10.1 COMPLIANCE WITH ETHICAL PRINCIPLES**

This study will be conducted in accordance with the spirit of the Declaration of Helsinki, the study protocol, the standards specified under the Pharmaceutical Affairs Act Article 80, Paragraph 2, GCP effective as of April 1, 1997 (Ministry of Health and Welfare Ordinance No. 28), and the related ministerial ordinances and notifications.

In addition, the study protocol, etc., will be periodically revised if necessary.

**10.2 INSTITUTIONAL REVIEW BOARD**

Prior to beginning the study, the IRB of the institution will examine the study protocol, CRF format, elements of the written informed consent form, and adequacy of study duration.

**10.3 INFORMED CONSENT**

**10.3.1 When and How to Obtain Informed Consent**

The principal investigator/subinvestigator will clarify the elements of 10.3.2 on the basis of the explanatory statement to the study participants 1 week prior to study drug administration and obtain their freely given written consent after verifying their understanding. The written informed consent form will be sealed or signed and personally dated by both the subject and the principal investigator/subinvestigator who will conduct the consent discussion. In addition to documenting this information in the CRF, the consent forms will also be submitted to the department assigned by the institution or attached to the medical records, and copies of the consent form (including the explanatory statement) will be provided to all subjects.

If information relevant to the subject`s willingness to continue participation in the study becomes available, the principal investigator/subinvestigator will inform the subjects in a timely manner. Upon ascertaining the subject’s continued interest, a written verification thereof will be dated and recorded.

**10.3.2 Elements of Informed Consent**

The explanatory statement (of the informed consent form) will be used to explain the following content of the study to the subjects.

(1) That the study involves research

(2) The purpose of the study

(3) The name, title, and contact information of the principal investigator

(4) The study procedures to be followed (such as the experimental aspects and selection criteria)

(5) The reasonably expected benefits and foreseeable risks or inconveniences to the subject (when there is no intended clinical benefit to the subject, the subject should be made aware of this)

(6) The expected duration of the subject’s participation in the trial

(7) That the subject’s participation in the trial is voluntary and that the subject may refuse to participate or withdraw from the study at any time without penalty or loss of benefits to which the subject is otherwise entitled

(8) That the monitor(s), auditor(s), IRB/IEC, and regulatory authority(ies) will be granted direct access to the subject’s original medical records for verification of the clinical study procedures and/or data, without violating the confidentiality of the subject, and that by sealing or signing the written informed consent form, the subject is authorizing such access

(9) That records identifying the subject will remain confidential if the results of the study are published

(10) The institution’s customer service number to contact to obtain further information regarding the study and rights of subjects and in the event of study-related injury

(11) The compensation and treatment available to subjects in the event of study-related injury

(12) The approximate number of subjects involved in the study

(13) That the subject will be informed in a timely manner if information relevant to the subject`s willingness to continue participation in the study becomes available

(14) The foreseeable circumstances and/or reasons under which the subject`s participation in the study may be terminated

(15) The anticipated expenses and reasons, if any, for the subject to participate in the study

(16) The anticipated prorated payment, if any, to the subject for participating in the study (agreement on amount, etc.)

(17) The subject’s responsibilities (including explanation of dietary restrictions)

**10.3.3 Approval and Revision of Informed Consent**

The principal investigator will prepare the written informed consent form and obtain approval from the IRB before the study is initiated.

The principal investigator must promptly notify the institution head/IRB in writing if new information on the safety of the study drug becomes available and implement appropriate measures. If the institution head/IRB deems it necessary to revise the consent form, revisions must be promptly made.

The principal investigator/subinvestigator will explain the revised consent form again to the subjects and obtain their signed voluntary consent relevant to their continued participation in the study.

The principal investigator/subinvestigator will document the date of the new consent in the CRF and provide copies of the newly sealed or signed and dated consent forms with the explanatory statement to the subjects.

**10.4 CONFIDENTIALITY**

Subject identification codes will be used to codify subjects in the CRF and protect their anonymity from third parties. Specifically, subject codes assigned during enrollment will be used in lieu of the subjects’ names and initials for identification and reference. Presentation and publication of study results must protect the subjects’ identities at all times.

**10.5 SUBJECT STIPEND**

Based on relevant institutional regulations or deliberation between the institution and sponsor, subjects will be compensated for participation (including transportation fees) to resolve any inconvenience incurred. The method of payment will be decided between the institution and sponsor.

**10.6 HEALTH DAMAGE COMPENSATION AND INSURANCE**

Medical treatment and other necessary interventions will be provided to subjects who incur study-related injuries. The sponsor will take precautions such as enrolling in insurance to indemnify against claims arising from the trial. If health damages occur and subjects are entitled to compensation as determined by the principal investigator and sponsor and if the institution and subjects request for compensation, the principal investigator and chief investigator, upon discussion, will immediately take the necessary steps in accordance with the Tsumura-GCP/SOP. In that case, the sponsor will cover all medical expenses, medical allowances for health damages for which hospitalization or higher level of treatment is required according to the benefits of the Relief System for Sufferers of Adverse Drug Reactions, and compensation based on the amount according to the Workmen’s Accident Compensation Insurance.

**10.7 STUDY COMPLETION OR PREMATURE TERMINATION AND SUSPENSION**

**10.7.1 Study Completion**

The principal investigator will prepare and submit the final report containing the elements outlined below to the institution head in a timely manner after the study is completed. The institution head will subsequently send a notification of study completion to the sponsor.

(1) Submission date of the final report

(2) Name and address of the sponsor

(3) Title of the study

(4) Names of the principal investigators (including every individual involved in the study)

(5) Study duration

(6) Number of subjects

(7) Summary of the study results

(8) Status of GCP compliance

**10.7.2 Criteria for Study Termination or Suspension**

The sponsor will terminate or suspend the entire study upon consultation with the principal investigator if any of the following events occur during the study.

(1) SAE(s) that undermines the continuation of the study

(2) Frequent AE(s) that undermines the continuation of the study

(3) The latest basic study results (including those from abroad) other than the results from the present study suggest serious implications for the subjects

(4) Termination or suspension of the study is decided for other reasons

**10.7.3 Procedures for Premature Termination or Suspension**

If the entire study is forced to be prematurely terminated or suspended due to any of the reasons stated above, the sponsor must promptly notify the reasons for termination or suspension in writing to the principal investigator and institution head/IRB.

In addition, the principal investigator must promptly inform the subjects, implement the necessary measures, document subjects’ study-related data in the CRF, and submit them to the sponsor.

**10.7.4 Procedures for Resuming the Study**

After suspension of the entire study, the institution head/IRB will consider continuation of the study. The principal investigator will be able to resume the study only if continuation of the entire study is deemed possible.

**11 PROTOCOL COMPLIANCE AND DEVIATION(S) OR CHANGE(S) AND AMENDMENT(S)**

**11.1 PROTOCOL COMPLIANCE**

The study will be executed in accordance with the study protocol approved by the principal investigator and sponsor.

**11.2 PROTOCOL DEVIATION(S) OR CHANGE(S)**

In general, deviations or changes to the study protocol by the principal investigator/subinvestigator are not permitted, except when medically necessary or authorized by the institution head in accordance with the IRB’s decision.

The principal investigator/subinvestigator will document all deviations from the protocol, provide detailed written explanation to the sponsor, and retain a copy of the original.

The principal investigator/subinvestigator may deviate or revise the protocol without prior written consent from the sponsor and approval from the IRB only to eliminate immediate hazards to the subjects or for compelling medical reasons.

The principal investigator will promptly provide notification to the sponsor, institution head, and IRB of any change that may significantly impact the study or increase the risk for subjects.

**11.3 PROTOCOL AMENDMENT(S)**

If protocol amendments are forced to be implemented after initiation of the study, the sponsor will notify the institution head and principal investigator of these changes. Depending on the extent of changes, the institution head will report or request review from the IRB and provide instructions to the principal investigator depending on the review. As evidence of agreement between the sponsor and principal investigator concerning the implemented amendments, both parties will seal, sign, and date the revised protocol or a surrogate document.

**12 DATA COLLECTION AND STORAGE**

**12.1 CASE REPORT FORM**

Upon completion of all clinical and laboratory evaluations, the principal investigator/subinvestigator will promptly generate the CRF, review the content, and seal or sign in the principal investigator’s section on the cover of the CRF.

The principal investigator will review the content and check the CRF prepared by the subinvestigator to ensure accuracy, and if acceptable, will seal or sign in the principal investigator’s section before submitting the CRF to the sponsor.

The seal in the CRF should be consistent with the one on the “List of Signatures and Seals (Form 4)” prepared beforehand.

**12.2 STORAGE OF RECORDS**

The personnel responsible for storage of records, as designated by the institution head, must retain the following essential documents for 3 years at the institution after formal discontinuation or completion of the study: informed consent documents, source data used for preparing the CRF (medical records, laboratory data, etc.), IRB’s deliberation documents and records, documents related to the study proposal and contract, the table of study drug administration, etc. If the records require transfer for any reason, the sponsor must be notified of the new address.

**13 SOURCE DOCUMENT VERIFICATION**

The principal investigator and the institution (the study site) will permit direct access to all study-related documents to provide source data for the IRB, for trial-related monitoring, for auditing by sponsors, and during inspection by the IRB and regulatory agencies.

**13.1 SOURCE DOCUMENT SPECIFICATIONS**

Prior to initiation of the study, the principal investigator and sponsor will prepare the “List of Source Documents (Form 5)” to jointly specify source documents that contain data for the CRF, storage area, and personnel responsible for storage.

However, for all comments, abnormal changes in laboratory values, AEs, ADRs, and causality assessment, the CRF will be used as the source document.

**13.2 PROCEDURE FOR DIRECT ACCESS**

Before initiating the study, the sponsor and principal investigator/institution will mutually agree on the procedures for directly accessing the source documents. (Refer to “Procedures for Direct Access to Source Documents”)

**13.3 EVALUATION AND HANDLING OF VERIFICATION OF RESULTS**

The principal investigator will provide written explanation of any discrepant results between the CRF data and other source documents to the sponsor, and retain a copy of the original.

Any confirmed deviation or noncompliance with the protocol will be promptly recorded and notified to the principal investigator by the sponsor. In addition, the sponsor will explicate appropriate protocol adherence to the principal investigator in order to secure his/her compliance.

**14 QUALITY CONTROL AND QUALITY ASSURANCE**

**14.1 QUALITY CONTROL**

In order to evaluate GCP and whether the quality of performance of both the statistical analysis departments and study drug administrator fulfill the necessary requirements, the sponsor’s study-related department will perform quality control in accordance with Tsumura-GCP/SOP.

**14.2 QUALITY ASSURANCE**

The sponsor’s GCP audit department will conduct a GCP audit according to the Tsumura-GCP/SOP audit schedule for evaluating the study’s compliance with the protocol, Tsumura-GCP/SOP, Tsumura GCP, and standard GCP. The GCP audit will be conducted at the institution and other study-related sites.

**15 PUBLICATION POLICY**

The results of the study may not be presented at academic conferences or published in medical journals without the consent of the sponsor.

**16 STUDY PERIOD**

September 2013–May 2014

**17 STUDY ORGANIZATIONS**

**17.1 SPONSOR**

**17.1.1 Sponsor**

Kampo Research Planning Department

Tsumura & Co.

2-17-11 Akasaka Minato-ku, Tokyo 107-8521, Japan

[Contact Information]

TEL: +813-6361-7184, FAX: +813-5574-6664

**17.1.2 Sponsor’s Study Organization**

The study organization of the sponsor is described in the “Study Organization of the Sponsor (Appendix 1).”

**17.1.3 Medical Advisor**

Professor Akio Inui

Psychosomatic Internal Medicine, Social and Behavioral Medicine

Kagoshima University Graduate School of Medical and Dental Sciences

8-35-1, Sakuragaoka, Kagoshima 890-8520, Japan

TEL: +81-99-275-5748, FAX: +81-99-275-5748

[Role of the Medical Advisor (Medical Expert)]

The medical expert is an appropriately qualified medical personnel who will be readily available to advise on study-related medical questions or problems. Specifically, the medical advisor will review the content of the AE reports received from the study department.

**17.2.1 Testing Facility for TJ-43 Constituents in Plasma**

(1) Shin Nippon Biomedical Laboratories, Ltd.

Pharmacokinetics and Bioanalysis Center

16-1, Minamiakasaka, Kainan-shi, Wakayama 642-0017, Japan

TEL: +81-73-483-8881, FAX: +81-73-483-7377

(2) Mitsubishi Chemical Medience Corporation

Testing and Research Center

1285 Kurisakicho Uto-shi, Kumamoto 869-0425, Japan

TEL: +81-964-23-5111, FAX: +81-964-23-5129

**17.2.2 Testing Facility for Exploratory Retrieval of Metabolites of TJ-43 Constituents in Plasma**

Tsumura & Co.

Tsumura Research Laboratories

3586 Yoshiwara, Amimachi Inashiki-gun, Ibaraki 300-1192, Japan

TEL: +81-29-889-3852, FAX: +81-29-889-3870

**17.3 TESTING FACILITY FOR TJ-43 CONSTITUENTS IN THE DRUG LOT**

Sumika Chemical Analysis Service, Ltd.

1-135, Kasugade-Naka 3-chome, Konohana-ku, Osaka 554-0022, Japan

TEL: +81-6-6466-5373, FAX: +81-6-8466-5493

**17.4 RESEARCH FACILITY AND PRINCIPAL INVESTIGATOR**

Research facility: Kochi Medical School Hospital

Oko-cho, Kohasu, Nankoku-shi, Kochi 783-8505, Japan

TEL: +81-88-866-5811

Principal Investigator: Professor Kazuhiro Hanazaki, M.D., Ph.D.

First Department of Surgery, Kochi Medical School Hospital

**18 REFERENCES**

1. Oka T, et al. Rikkunshi-to attenuates adverse gastrointestinal symptoms induced by fluvoxamine. Biopsychosoc Med. 2007; Nov 15, 1: 21.
2. Harasawa S, et al. Multi-centered joint post-marketing clinical trial of Rikkunshito (TJ-43) for dysmotility-like dyspepsia. Igaku no Ayumi. 1998; 187; No. 3: 207-229.
3. Suzuki H, et al. Japanese herbal medicine in functional gastrointestinal disorders. Neurogastroenterol Motil 2009; 21: 688-696.
4. Tominaga K, et al. Rikkunshito improves symptoms in PPI-refractory GERD patients: a prospective, randomized, multicenter trial in Japan. J Gastroenterol. 2011; Nov 15.
5. Kawahara H, et al. Effects of rikkunshito on the clinical symptoms and esophageal acid exposure in children with symptomatic gastroesophageal reflux. Pediatr Surg Int. 2007; 23(10): 1001-1005.
6. Kusunoki H, et al. Efficacy of Rikkunshito, a traditional Japanese medicine (Kampo), in treating functional dyspepsia. Intern Med. 2010; 49(20): 2195-2202.
7. Kobayashi S, et al. Diagnosis and treatment. 1996; 84(2): 305.
8. Tatsuta M, et al. Effect of treatment with liu-jun-zi-tang (TJ-43) on gastric emptying and gastrointestinal symptoms in dyspeptic patients. Aliment Pharmacol Ther. 1993; 7(4): 459-462.
9. Takahashi T, et al. Effect of rikkunshito, a Chinese herbal medicine, on stasis in patients after pylorus-preserving gastrectomy. World J Surg. 2009; 33(2): 296-302.
10. Kido T, et al. Effects of rikkunshi-to, a traditional Japanese medicine, on the delay of gastric emptying induced by N(G)-nitro-L-arginine. J Pharmacol Sci. 2005; 98(2): 161-167.
11. Takeda H, et al. Rikkunshito, an herbal medicine, suppresses cisplatin-induced anorexia in rats via 5-HT2 receptor antagonism. Gastroenterology. 2008; 134(7): 2004-2013.
12. Fujitsuka N, et al. Selective serotonin reuptake inhibitors modify physiological gastrointestinal motor activities via 5-HT2c receptor and acyl ghrelin. Biol Psychiatry. 2009; 65(9): 748-759.
13. Yakabi K, et al. Reduced ghrelin secretion in the hypothalamus of rats due to cisplatin-induced anorexia. Endocrinology. 2010; 151(8): 3773-3782.
14. Matsumura T, et al. The traditional Japanese medicine Rikkunshito increases the plasma level of ghrelin in humans and mice. J Gastroenterol. 2010; 45(3): 300-307.
15. Fujitsuka N, et al. Potentiation of ghrelin signaling attenuates cancer anorexia-cachexia and prolongs survival. Translational Psychiatry. 2001; 1: e23.
16. Zhao WJ, et al. Determination of glycyrrhetic acid in human plasma by HPLC-MS method and investigation of its pharmacokinetics. J Clin Pharm Ther. 2008; 33(3): 289-294.
17. Ding L, et al. Determination of glycyrrhetic acid in human plasma by LC-ESI-MS. J Pharm Biomed Anal. 2006; 240(3): 758-762.
18. Makino T, et al. Down-regulation of a hepatic transporter Mrp2 is involved in alteration of pharmacokinetics of glycyrrhizin and its metabolites in a rat model of chronic liver injury. Drug Metab Dispos. 2008; 36(7): 1438-1443.
19. Company Document of Tsumura
20. Company Document of Tsumura

**19 STATISTICIAN AND MEDICAL ADVISOR (MEDICAL EXPERT) VERIFICATION**

[Ver. 1.1 Prepared on June 2, 2014]

Statistician Month Day Year

Medical Advisor Month Day Year

**20 STATEMENT OF COMPLIANCE WITH THE PROTOCOL**

Statement of Agreement

The seals and signatures below constitute the agreement and approval of this protocol “Post-marketing clinical study (TJ-43-4-2) of Rikkunshito (TJ-43)” (Ver. 1.1) and provide the necessary assurances that this trial will be conducted in compliance with the protocol.

Principal Investigator Month Day 2014

Research facility Kochi Medical School Hospital

Affiliation/Title First Department of Surgery, Professor

Name Kazuhiro Hanazaki seal

Study Monitor Month Day 2014

Tsumura & Co.

Head of Kampo Research Planning Department

Name Hideki Mitsuwa seal
