# Supplementary material for: Pharmacokinetic Profiles of Active Ingredients and Its Metabolites Derived from Rikkunshito, a Ghrelin Enhancer, in Healthy Japanese Volunteers: A Cross-Over, Randomized Study
Source: PLoS One. 2015 Jul 17;10(7):e0133159. doi: 10.1371/journal.pone.0133159 (PMC4506051; doi:10.1371/journal.pone.0133159)
Supplement: S1 Appendix — (DOCX) [file pone.0133159.s003.docx]

Analysis of 32 ingredients in plasma and urine

For quantification of atractylodin, 25 µL of standard solution or methanol was added to 500 µL of plasma or urine samples, followed by mixing well. For urine, 500 µL of blank plasma was added to the solutions. Diethyl ether (3 mL) was added to the solutions followed by shaking (horizontal direction, 300 r/min, 10 min) and centrifugation (1,910 ×*g*, 5 min, 4°C). The supernatants were collected and dried at 25°C under a stream of nitrogen gas. Then, 200 μL of acetonitrile was added to the dried samples followed by mixing and sonicating. The solutions were then filtrated (0.22 µm; Millipore Corporation, Billerica, MA) and centrifuged (9,100 ×*g*, 2 min, 4°C) followed by injection into a gas chromatography–mass spectrometry instrument. The gas chromatography–mass spectrometry system comprised an HP6890 series gas chromatograph connected to a HP5973N mass selective detector (Agilent Technologies, Santa Clara, CA). The GC column was a HP-1 30 mm × 0.25 mm i.d. capillary of 0.25-μm film thickness (Agilent Technologies). The temperature was set at 100°C for 5 min and then raised to 250°C at 15°C per min. The final temperature of 250°C was maintained for 5 min. The carrier gas was helium with a capillary splitless injector. The injection port temperature was 300°C. Sample injection quantity was 1 μL. Atractylodin was quantified by selected ion monitoring transitions at 182 to 152 *m/z*.

For quantification of synephrine, 5 µL of standard solution or methanol was added to 100 µL plasma or urine samples followed by mixing. Ten microliters of erythromycin [internal standard (IS)] solution was added to the solutions followed by mixing and centrifugation (20,400 ×*g*, 5 min, 4°C). The supernatants were loaded onto an Ostro 96-well plate (Waters) and centrifuged (1500 ×*g*, 3 min, 4°C) followed by injection into the LC–MS/MS.

For quantification of 30 ingredients other than atractylodin and synephrine, 25 µL of standard solution or methanol was added to 500 µL plasma or urine samples followed by mixing. Atractylenolide III or digoxin (IS) solution (50 μL) was added to the solutions followed by mixing. For urine, 400 µL of blank plasma was added to the solutions. Ammonium acetate solution (100 mM, 100 or 400 μL) was added to the solutions followed by mixing. For enzymatic treatment, plasma and urine samples were incubated with β-glucuronidase (100 units in 100 mM ammonium acetate) for 2 h at 37°C. A solid-phase cartridge (OASIS HLB, 30 mg/1cc, Waters) was conditioned with methanol and 10 mM ammonium acetate solution, and the samples were loaded onto the cartridge. The cartridge was washed water : methanol (9:1, v/v), and 1.5 mL of methanol and 30 µL of propylene glycol were added to the cartridge to elute the analytes. The mixtures were dried at 25°C under a stream of nitrogen gas. Furthermore, 100 μL of 10 mM ammonium acetate : methanol (8:2, v/v) was added to the dried samples followed by mixing and sonication. The solutions were filtered (0.22 µm) and followed by injection into the LC–MS/MS system. This system comprised an LC-20A system (Shimadzu, Kyoto, Japan) connected to an API5000 triple quadrupole mass spectrometer fitted with a TurboIonSpray electrospray ionization interface (AB Sciex). Those analytical conditions are summarized in S2 Table and S3 Table.

Analysis of nine ingredients derived from rikkunshito in plasma

For quantification of nobiletin, heptamethoxyflavone, pachymic acid, and 18β-glycyrrhetinic acid, 30 µL of standard solution or 50% methanol, and 50 µL of (±)-warfarin-d5 (IS) solution were added to 300 µL each of samples followed by 1 mL of 10% methanol solution. A solid-phase cartridge (OASIS HLB, 60 mg/3cc) was conditioned with methanol and 10% methanol solution. The samples were loaded onto the cartridge. The cartridge was washed with 20% methanol solution, and 1 mL of methanol was added to elute the analytes. Propylene glycol solution (5% (v/v), 300 μL) was added to the eluate and the solution was evaporated to dryness under a stream of nitrogen gas at 40°C or below. To the residue, 100 μL of methanol:10 mM ammonium acetate (3:7, v/v) were added followed by mixing. The solutions were injected onto the LC–MS/MS system described above.

For quantification of naringenin, isoliquiritigenin, and liquiritigenin, 30 µL of standard solution or 50% methanol solution and 50 µL of (±)-warfarin-d5 (IS) solution were added to 300 µL each of samples, after which 150 µL of 0.1% hydrochloric acid solution was added followed by mixing. *t*-Butyl methyl ether (2 mL) was added to the solutions, which were shaken for 10 min. The solutions were centrifuged (2,370 ×*g*, 10 min, 4°C), and frozen in a dry ice/acetone bath. The upper (organic) layer was transferred to clean polypropylene tubes. Propylene glycol solution (5% (v/v), 300 μL) was added to the organic layer, and the solution was evaporated to dryness under a stream of nitrogen gas at ≤40°C. To the residue 100 μL of methanol:10 mM ammonium acetate (3:7, v/v) was added followed by mixing. The solutions were injected onto an LC–MS/MS comprising a LC-30A system (Shimadzu) connected to a Triple Quad 6500 triple quadrupole mass spectrometer fitted with a TurboIonSpray electrospray ionization interface (AB Sciex).

For quantification of atractylodin, 100 μL of standard solution or methanol was added to 500 μL each of samples, and the resulting solution was mixed and centrifuged (15,000 ×*g*, 4°C, and 10 min). A solid-phase plate (Oasis HLB μElution plate, Waters) was conditioned with methanol and water, and the samples were loaded onto the cartridge. The cartridge was washed with methanol:water (4:1, v/v), and 100 μL of acetonitrile was added to elute the analyte. After addition of 20 μL of atractylenolide III (IS) solution followed by mixing, the solutions were injected into an LC–MS/MS system comprising a LC-20AD system (Shimadzu) connected to an API5000 triple quadrupole mass spectrometer fitted with an atmospheric pressure chemical ionization interface (AB Sciex).

For quantification of atractylodin metabolite (atractylodin carboxylic acid), 400 µL of acetonitrile and 10 µL of atractylenolide III (IS) solution were added to 100 µL each of study samples or calibration curve samples, and the resulting solution was mixed and centrifuged (13,800 ×*g*, 5 min). The supernatant was collected and centrifuged under vacuum to remove the solvent. After addition of 20% (v/v) methanol solution and mixing, the solutions were injected into an LC–MS/MS system comprising an Agilent 1260 series (Agilent Technologies) connected to a QTRAP5500 triple quadrupole mass spectrometer fitted with a TurboIonSpray electrospray ionization interface (AB Sciex). S4 Table and S5 Table show the LC–MS/MS conditions for analyzing the nine ingredients derived from rikkunshito.

Measurement of ingredients in rikkunshito formulation

Rikkunshito (500 mg, lot H05142) was suspended in 25 mL of acetonitrile/purified water (5:5, v/v) and ultrasonicated for 15 min. The sonicated solution was centrifuged at 1,910 ×*g* and 4°C for 15 min, and the supernatant was collected. The residue was re-extracted with 25 mL of methanol/purified water (5:5, v/v). The first and second supernatants were combined, filtered (0.22 mm), and diluted with extracted solution as the test substance solution. Sixteen ingredients (glycyrrhizic acid, isoliquiritin, liquiritin, liquiritigenin, liquiritin apioside, isoliquiritin apioside, hesperidin, narirutin, naringin, atractylodin, nobiletin, heptamethoxyflavone, pachymic acid, naringenin, isoliquiritigenin, and 18β-glycyrrhetinic acid) in the solution were measured by HPLC and LC–MS/MS. LC-10A was used for atractylodin analysis at a UV detection wavelength of 340 nm. The LC–MS/MS system comprised an LC-20A system connected to an API5000 triple quadrupole mass spectrometer fitted with an atmospheric pressure chemical ionization interface. S6 Table and S7 Table show the LC–MS/MS conditions for analysis of 16 ingredients.
