# Supplementary figures and images for: Pharmacokinetic Profiles of Active Ingredients and Its Metabolites Derived from Rikkunshito, a Ghrelin Enhancer, in Healthy Japanese Volunteers: A Cross-Over, Randomized Study
Source: PLoS One. 2015 Jul 17;10(7):e0133159. doi: 10.1371/journal.pone.0133159 (PMC4506051; doi:10.1371/journal.pone.0133159)

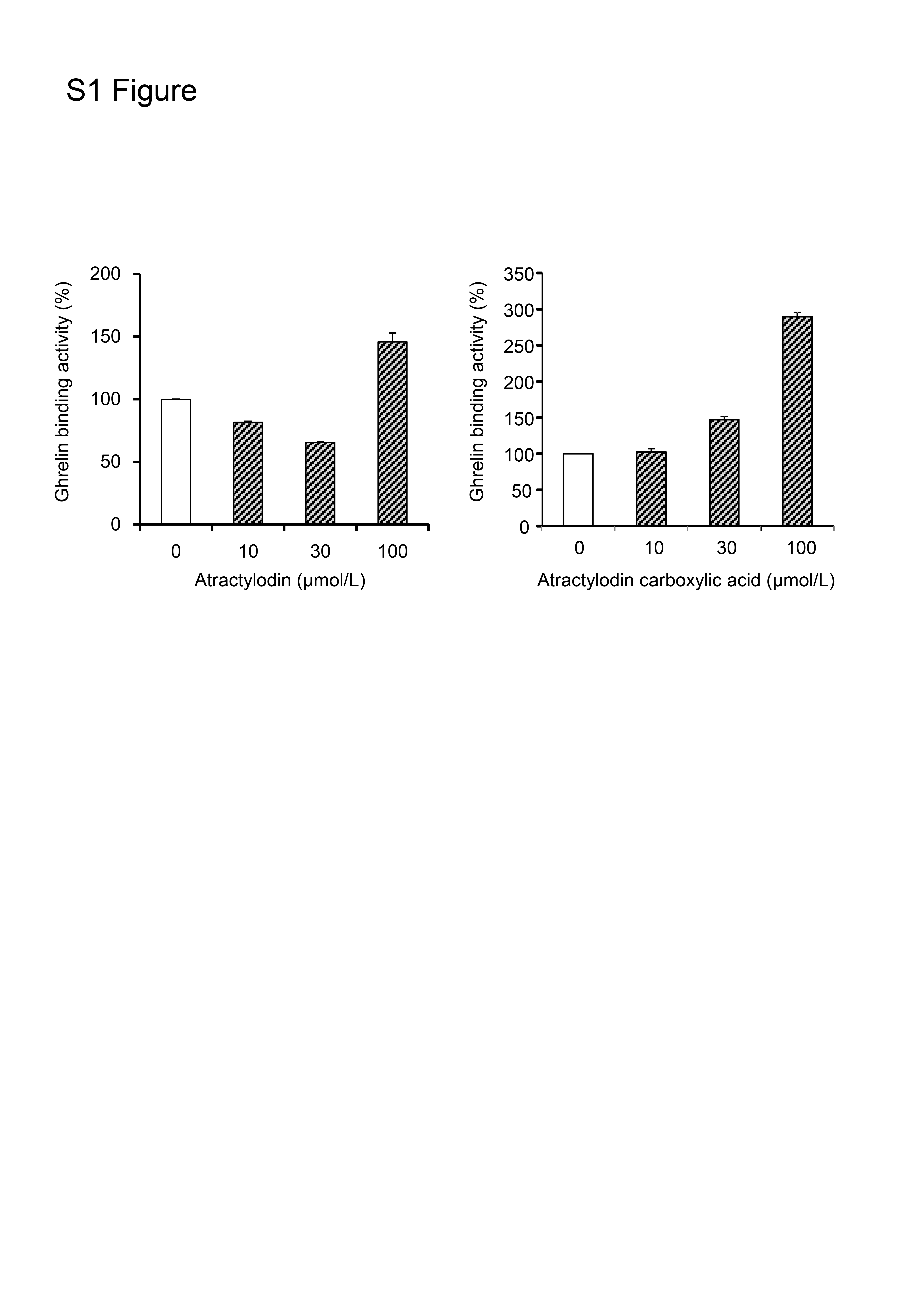

Supplement: S1 Fig — Radioligand binding was performed using GSH-R-expressing cells (n = 3). (TIF) [file pone.0133159.s004.tif]
