# Supplementary material for: Pharmacokinetic Profiles of Active Ingredients and Its Metabolites Derived from Rikkunshito, a Ghrelin Enhancer, in Healthy Japanese Volunteers: A Cross-Over, Randomized Study
Source: PLoS One. 2015 Jul 17;10(7):e0133159. doi: 10.1371/journal.pone.0133159 (PMC4506051; doi:10.1371/journal.pone.0133159)
Supplement: S1 Table — (DOCX) [file pone.0133159.s005.docx]

**S1 Table. Typical 32 ingredients included in rikkunshito.**

| Herbal medicines | Compound |  | Herbal medicines | Compound |
| --- | --- | --- | --- | --- |
| *Zingiberis Rhizoma* | [6]-Gingerol |  | *Glycyrrhizae radix* | Liquiritin apioside |
|  | [8]-Gingerol |  |  | Liquiritin |
|  | [10]-Gingerol |  |  | Isoliquiritigenin |
|  | [6]-Shogaol |  |  | Glycycoumarin |
|  | [8]-Shogaol |  |  | 18β-Glycyrrhetinic acid |
| *Atractylodis Lanceae Rhizoma* | Atractylodin |  |  | Glycyrrhetinic acid 3-*O*-glucuronide |
| *Ginseng Radix* | Ginsenoside Rb_1_ |  | *Citri unshiu pericarpium* | Hesperidin |
|  | Ginsenoside Rb_2_ |  |  | Hesperetin |
|  | Ginsenoside Rc |  |  | Narirutin |
|  | Ginsenoside Rd |  |  | Nobiletin |
|  | Ginsenoside Re |  |  | Tangeretin |
|  | Ginsenoside Rf |  |  | Heptamethoxyflavone |
|  | Ginsenoside Rg_1_ |  |  | Synephrine |
|  | Ginsenoside Rg_2_ |  |  | Naringin |
| *Poria* | Pachymic acid |  |  | Naringenin |
| *Zizyphi fructus* | Oleanolic acid |  | *Pinelliae Tuber* | PTH-15 |
