# Supplementary material for: Pharmacokinetic Profiles of Active Ingredients and Its Metabolites Derived from Rikkunshito, a Ghrelin Enhancer, in Healthy Japanese Volunteers: A Cross-Over, Randomized Study
Source: PLoS One. 2015 Jul 17;10(7):e0133159. doi: 10.1371/journal.pone.0133159 (PMC4506051; doi:10.1371/journal.pone.0133159)
Supplement: S2 Table — (DOCX) [file pone.0133159.s006.docx]

**S2 Table. Methods of LC-MS/MS for analysis of plasma and urine samples: Ion parameters of rikkunshito ingredients and internal standards.**

| Compound | Q1Mass (*m/z*) | Q3Mass (*m/z*) | Polarity | LC methods ID |
| --- | --- | --- | --- | --- |
| Synephrine | 168 | 150 | Positive | 1-1 |
| Erythromycin (IS) | 735 | 158 | Positive | 1-1 |
| 18β-Glycyrrhetinic acid | 471 | 149 | Positive | 1-2 |
| Glycyrrhetinic acid 3-*O*-glucuronide | 648 | 453 | Positive | 1-2 |
| Nobiletin | 403 | 373 | Positive | 1-2 |
| Tangeretin | 373 | 343 | Positive | 1-2 |
| Heptamethoxyflavone | 433 | 403 | Positive | 1-2 |
| Atractylenolide III (IS) | 249 | 231 | Positive | 1-2 |
| [6]-Gingerol | 293 | 99 | Negative | 1-3 |
| [8]-Gingerol | 321 | 127 | Negative | 1-3 |
| [10]-Gingerol | 349 | 155 | Negative | 1-3 |
| [6]-Shogaol | 275 | 139 | Negative | 1-3 |
| [8]-Shogaol | 303 | 167 | Negative | 1-3 |
| Ginsenoside Rb_1_ | 1108 | 179 | Negative | 1-3 |
| Ginsenoside Rb_2_ | 1078 | 149 | Negative | 1-3 |
| Ginsenoside Rc | 1078 | 191 | Negative | 1-3 |
| Ginsenoside Rd | 946 | 161 | Negative | 1-3 |
| Ginsenoside Re | 946 | 119 | Negative | 1-3 |
| Ginsenoside Rf | 800 | 161 | Negative | 1-3 |
| Ginsenoside Rg_1_ | 800 | 638 | Negative | 1-3 |
| Ginsenoside Rg_2_ | 784 | 476 | Negative | 1-3 |
| Pachymic acid | 528 | 465 | Negative | 1-3 |
| Liquiritin apioside | 550 | 255 | Negative | 1-3 |
| Liquiritin | 417 | 255 | Negative | 1-3 |
| Isoliquiritigenin | 255 | 119 | Negative | 1-3 |
| Glycycoumarin | 367 | 309 | Negative | 1-3 |
| Hesperidin | 609 | 301 | Negative | 1-3 |
| Hesperetin | 301 | 164 | Negative | 1-3 |
| Narirutin | 579 | 271 | Negative | 1-3 |
| Naringin | 579 | 271 | Negative | 1-3 |
| Naringenin | 271 | 119 | Negative | 1-3 |
| PTH-15 | 563 | 383 | Negative | 1-3 |
| Oleanolic acid | 455 | 407 | Negative | 1-3 |
| Digoxin (IS) | 780 | 651 | Negative | 1-3 |

IS; internal standard.
