# Supplementary material for: Pharmacokinetic Profiles of Active Ingredients and Its Metabolites Derived from Rikkunshito, a Ghrelin Enhancer, in Healthy Japanese Volunteers: A Cross-Over, Randomized Study
Source: PLoS One. 2015 Jul 17;10(7):e0133159. doi: 10.1371/journal.pone.0133159 (PMC4506051; doi:10.1371/journal.pone.0133159)
Supplement: S3 Table — (DOCX) [file pone.0133159.s007.docx]

**S3 Table. Methods of LC-MS/MS for analysis of plasma and urine samples: HPLC conditions for analyzing rikkunshito ingredients.**

| Methods ID | HPLC condition | |
| --- | --- | --- |
| 1-1 | Column: | HALO HILIC (2.1 mm I.D., × 30 mm L., 2.7-μm particle size; Advanced materials technology, Inc., , Wilmington, DE) |
|  | Mobile phase: | (A) 20 mM ammonium acetate (pH 4.0), (B) acetonitrile |
|  | Gradient elution program  (%B in A) | isocratic at 90% (0–4 min) |
|  | flow rate | 0.8 mL/min; |
|  | column temperature | 40°C |
|  | injection volume | 4 μL |
| 1-2 | Column: | shim-pack XR-ODS II (2.0 mm I.D., × 100 mm L., 2.2-μm particle size; Shimadzu GLC Ltd., Tokyo, Japan) |
|  | Mobile phase | (A) 10 mM ammonium acetate, (B) methanol |
|  | Gradient elution program  (%B in A) | 0.01–0.50 min, 20%; 0.50–2.50 min, 20–40%; 2.50–17.00 min, 40–85%; 17.00–30.00 min, 85–95%; 30.00–34.00 min, 95%; 34.10–40.00 min, 20% |
|  | flow rate | 0.2 mL/min |
|  | column temperature | 40°C |
|  | injection volume | 10 μL |
| 1-3 | Column: | shim-pack XR-ODS II (2.0 mm I.D., × 100 mm L., 2.2-μm particle size; Shimadzu GLC Ltd., Tokyo, Japan) |
|  | Mobile phase | (A) 10 mM ammonium acetate, (B) methanol |
|  | Gradient elution program (%B in A) | 0.01–0.50 min, 20%; 0.50–2.50 min, 20–40%; 2.50–17.00 min, 40–85%; 17.00–30.00 min, 85–95%; 30.00–34.00 min, 95%; 34.10–40.00 min, 20% |
|  | flow rate | 0.2 mL/min |
|  | column temperature | 40°C |
|  | injection volume | 30 μL |
