# Supplementary material for: Pharmacokinetic Profiles of Active Ingredients and Its Metabolites Derived from Rikkunshito, a Ghrelin Enhancer, in Healthy Japanese Volunteers: A Cross-Over, Randomized Study
Source: PLoS One. 2015 Jul 17;10(7):e0133159. doi: 10.1371/journal.pone.0133159 (PMC4506051; doi:10.1371/journal.pone.0133159)
Supplement: S4 Table — (DOCX) [file pone.0133159.s008.docx]

**S4 Table. Methods of LC-MS/MS for analysis of plasma samples: Ion parameters of 9 ingredients derived from rikkunshito and internal standards.**

| Compound | Q1Mass (*m/z*) | Q3Mass (*m/z*) | Polarity | LC methods ID |
| --- | --- | --- | --- | --- |
| Atractylodin | 183.0 | 153.0 | Positive | 2-1 |
| Atractylenolide III (IS) | 249.0 | 231.0 | Positive | 2-1 |
| Atractylodin carboxylic acid | 210.8 | 141.0 | Negative | 2-2 |
| Atractylenolide III (IS) | 247.0 | 203.0 | Negative | 2-2 |
| Pachymic acid | 546.4 | 451.3 | Positive | 2-3 |
| Heptamethoxyflavone | 433.6 | 403.0 | Positive | 2-3 |
| Nobiletin | 403.6 | 373.0 | Positive | 2-3 |
| 18β-Glycyrrhetinic acid | 471.8 | 189.2 | Positive | 2-3 |
| Warfarin-d5 (IS) | 314.1 | 163.1 | Positive | 2-3 |
| Naringenin | 271.0 | 151.0 | Negative | 2-4 |
| Liquiritigenin | 255.0 | 135.0 | Negative | 2-4 |
| Isoliquiritigenin | 255.1 | 119.0 | Negative | 2-4 |
| Warfarin-d5 (IS) | 312.1 | 161.0 | Negative | 2-4 |

IS; internal standard,
