# Supplementary material for: Pharmacokinetic Profiles of Active Ingredients and Its Metabolites Derived from Rikkunshito, a Ghrelin Enhancer, in Healthy Japanese Volunteers: A Cross-Over, Randomized Study
Source: PLoS One. 2015 Jul 17;10(7):e0133159. doi: 10.1371/journal.pone.0133159 (PMC4506051; doi:10.1371/journal.pone.0133159)
Supplement: S5 Table — (DOCX) [file pone.0133159.s009.docx]

**S5 Table. Methods of LC-MS/MS for analysis of plasma samples: Conditions of HPLC conditions for analyzing 9 ingredients derived from rikkunshito.**

| Methods ID | HPLC condition | |
| --- | --- | --- |
| 2-1 | Column | CAPCELLPAK C18 MGIII (150 × 4.6 mm I.D., 5.0-μm particle size; Shiseido Co., Ltd., Tokyo, Japan) |
|  | Mobile phase | (A) 10 mM ammonium acetate, (B) acetonitrile |
|  | Gradient elution program (% B in A) | isocratic at 80% (0–10 min) |
|  | flow rate | 0.8 mL/min |
|  | column temperature | 40°C |
|  | injection volume | 40 μL |
| 2-2 | Column: | CAPCELL CORE ADME (100 × 2.1 mm I.D., 2.7-μm particle size; Shiseido Co., Ltd.) |
|  | Mobile phase | (A) 0.2 vol % acetic acid, (B) acetonitrile containing 0.2 vol % acetic acid |
|  | Gradient elution program (% B in A) | 0.0–1.0 min, 40%; 1.0–8.0 min, 40–70%; 8.0–11.0 min, 70%; 11.1–17.0 min, 40% |
|  | flow rate | 0.2 mL/min |
|  | column temperature | 40°C |
|  | injection volume | 5 μL |
| 2-3 | Column: | Develosil ODS-UG-3 (100 × 2.0 mm I.D., 3.0-μm particle size; Nomura Chemical Co., Ltd., Aichi, Japan) |
|  | Mobile phase | (A) 10 mM ammonium acetate, (B) methanol |
|  | Gradient elution program (% B in A) | 0.01–0.50 min, 20%; 0.50–1.00 min; 20–40%; 1.00–8.00 min, 40–85%; 8.00–14.00 min, 85–95%; 14.01–17.00 min, 20% |
|  | flow rate | 0.4 mL/min |
|  | column temperature | 40°C |
|  | injection volume | 30 μL |
| 2-4 | Column | Develosil C30-UG-3 (150 × 2.0 mm I.D., 3.0-μm particle size; Nomura Chemical Co., Ltd.) |
|  | Mobile phase | (A) 10 mM ammonium acetate , (B) methanol |
|  | Gradient elution program (% B in A) | 0.01–1.00 min, 20%; 1.00–14.00 min, 20–40%; 14.01–17.00 min, 65%; 17.01–20.00 min, 20% |
|  | flow rate | 0.4 mL/min |
|  | column temperature | 40°C |
|  | injection volume | 30 μL |
