# Supplementary material for: Pharmacokinetic Profiles of Active Ingredients and Its Metabolites Derived from Rikkunshito, a Ghrelin Enhancer, in Healthy Japanese Volunteers: A Cross-Over, Randomized Study
Source: PLoS One. 2015 Jul 17;10(7):e0133159. doi: 10.1371/journal.pone.0133159 (PMC4506051; doi:10.1371/journal.pone.0133159)
Supplement: S6 Table — (DOCX) [file pone.0133159.s010.docx]

**S6 Table. Methods of LC-MS/MS for analysis of rikkunshito formulation: Ion parameters of rikkunshito ingredients, and internal standards.**

| Compound | Q1Mass (*m/z*) | Q3Mass (*m/z*) | Polarity | LC methods ID |
| --- | --- | --- | --- | --- |
| 18β-Glycyrrhetinic acid | 471 | 149 | Positive | 3-1 |
| Nobiletin | 403 | 373 | Positive | 3-1 |
| Heptamethoxyflavone | 433 | 403 | Positive | 3-1 |
| Glycyrrhizic acid | 824 | 453 | Positive | 3-1 |
| Atractylenolide III (IS) | 249 | 231 | Positive | 3-1 |
| Pachymic acid | 528 | 465 | Negative | 3-1 |
| Isoliquiritigenin | 255 | 119 | Negative | 3-1 |
| Naringenin | 271 | 119 | Negative | 3-1 |
| Digoxin (IS) | 780 | 651 | Negative | 3-1 |
| Isoliquiritin | 417 | 255 | Negative | 3-2 |
| Liquiritin | 417 | 255 | Negative | 3-2 |
| Liquiritigenin | 255 | 119 | Negative | 3-2 |
| Liquiritin apioside | 549 | 255 | Negative | 3-2 |
| Isoliquiritin apioside | 549 | 255 | Negative | 3-2 |
| Hesperidin | 609 | 301 | Negative | 3-2 |
| Narirutin | 579 | 271 | Negative | 3-2 |
| Naringin | 579 | 271 | Negative | 3-2 |
| Digoxin (IS) | 780 | 649 | Negative | 3-2 |
