# Supplementary material for: Pharmacokinetic Profiles of Active Ingredients and Its Metabolites Derived from Rikkunshito, a Ghrelin Enhancer, in Healthy Japanese Volunteers: A Cross-Over, Randomized Study
Source: PLoS One. 2015 Jul 17;10(7):e0133159. doi: 10.1371/journal.pone.0133159 (PMC4506051; doi:10.1371/journal.pone.0133159)
Supplement: S7 Table — (DOCX) [file pone.0133159.s011.docx]

**S7 Table. Methods of HPLC and LC-MS/MS for analysis of rikkunshito formulation: Conditions of HPLC conditions for analyzing rikkunshito ingredients.**

| Methods ID | HPLC condition | |
| --- | --- | --- |
| Analysisfor atractylodin | Column | Inertsil ODS-3 (50 mmL × 4.6 mm I.D., 3.0-μm particle size; GL Sciences Inc., Tokyo, Japan) |
|  | Mobile phase | (A) purified water, (B) acetonitrile |
|  | Gradient elution program (% B in A) | 0.01–3.00 min, 50%; 3.00–5.00 min, 50–95%; 5.00–10.00 min, 95%; 10.01–12.00 min, 50% |
|  | flow rate | 1.0 mL/min |
|  | column temperature | 40°C |
|  | injection volume | 50 μL |
| 3-1 | Column: | SUMIPAX ODS Z-CLUE (50 × 2.0 mm I.D., 3.0-μm particle size; Sumika Chemical Analysis Service Ltd.) |
|  | Mobile phase | (A) 10 mM ammonium acetate, (B) methanol |
|  | Gradient elution program (% B in A) | 0.01–3.00 min, 40–95%; 3.00–5.50 min, 95%; 5.51–7.00 min, 40% |
|  | flow rate | 0.35 mL/min |
|  | column temperature | 40°C |
|  | injection volume | 10 μL |
| 3-2 | Column | SUMIPAX ODS Z-CLUE (50 × 2.0 mm I.D., 3.0-μm particle size) |
|  | Mobile phase | (A) 10 mM ammonium acetate, (B) methanol |
|  | Gradient elution program (% B in A) | 0.01–0.50 min, 20%; 0.50–2.00 min, 20–30%; 2.00–6.50 min, 30%; 6.50–9.00 min, 30–50%; 9.00–10.00 min, 50–90%; 10.00–12.00 min, 90%; 12.01–15.00 min, 20% |
|  | flow rate | 0.35 mL/min |
|  | column temperature | 40°C |
|  | injection volume | 5 μL |
