# Supplementary material for: Pharmacokinetic Profiles of Active Ingredients and Its Metabolites Derived from Rikkunshito, a Ghrelin Enhancer, in Healthy Japanese Volunteers: A Cross-Over, Randomized Study
Source: PLoS One. 2015 Jul 17;10(7):e0133159. doi: 10.1371/journal.pone.0133159 (PMC4506051; doi:10.1371/journal.pone.0133159)
Supplement: S8 Table — (DOCX) [file pone.0133159.s012.docx]

**S8 Table. Validation items and results of 8 ingredients derived from rikkunshito.**

1. Atractylodin

| Validation items | Results | |
| --- | --- | --- |
| Selectivity | Ratio | Atractylodin: 0.0% |
|  |  | IS: 0.0% |
| Recovery  (0.600, 2.00, and 16.0 ng/mL) | Atractylodin | 54.1% to 57.0% |
| Intra-day reproducibility  (0.600, 2.00, and 16.0 ng/mL) | Relative error (RE; %) | -1.3% to 8.0% |
|  | Coefficient of variation (CV; %) | 4.3% to 4.4% |
| Inter-day reproducibility  (0.600, 2.00 and 16.0 ng/mL) | RE (%) | 4.4% to 10.2% |
|  | CV (%) | 7.4% to 8.2% |
| Calibration curve  (0.200, 0.600, 1.00, 2.00, 10.0, 16.0, and 20.0 ng/mL) | Correlation coefficient (*r*) | 0.9968 to 0.9975 |
|  | RE (%) | LLOQ: -3.0% to -1.5% |
|  |  | Others: -9.4% to 10.0% |
| Stability in blood (1.00 ng/mL)  At room temperature for 2 h | Remaining ratio (RR) | 90.3% |
| Stability in blood (1.00 ng/mL)  On ice for 2 h | RR | 93.8% |
| Short-term stability (0.600 and 16.0 ng/mL)  In a deep freezer (acceptable range: −90.4°C to −69.5°C) for 4 and 24 h | RR | 4 h: 95.6% and 101.9% |
|  |  | 24 h: 101.5% and 102.9% |
| Post-preparative stability (0.600 and 16.0 ng/mL)  In an autosampler set at 4°C for 24 and 48 h | RE | 24 h: -3.8% and 9.0% |
|  |  | 48 h: 4.0% and 8.7% |
| Freeze-thaw stability (0.600 and 16.0 ng/mL)  In a deep freezer (acceptable range: −90.4°C to −69.5°C) after 3 and 2 cycles of freeze-thaw | RR | 3 cycles: 115.1% and 133.6% |
|  |  | 2 cycles: 109.3% and 122.6% |
| Long-term stability (0.600 and 16.0 ng/mL)  In a deep freezer (acceptable range: −90.4°C to −69.5°C) for 14, 30, 57, and 120 days | RR | 15 days: 98.4% and 98.6% |
|  |  | 29 days: 88.7% and 98.6% |
|  |  | 57 days: 101.4% and 109.4 |
|  |  | 169 days: 95.2% and 102.8% |
| Dilution integrity (100 ng/mL)  (10-fold dilution) | RE | 1.0% |
|  | CV | 5.0% |
| Matrix effect | CV | Matrix factor: 4.7% |
|  |  | IS-normalized matrix factor: 5.7% |
| Carry-over | Ratio | Atractylodin: 0.0% |
|  |  | IS: 0.0% |
| Short-term stability of standard solution (atractylodin; 5000 and 1.00 ng/mL, IS; 2000 ng/mL)  At room temperature for 24 h | RR | Atractylodin: 106.7% and 104.6% |
|  |  | IS: 100.0% |
| Long-term stability of standard solution (IS; 2000 ng/mL)  In a refrigerator (acceptable range: 0.5°C to 7.4°C) for 4 weeks | RR | IS: 93.4% |

continued

1. Pachymic acid

| Validation items | Results | |
| --- | --- | --- |
| Selectivity | Ratio | Pachymic acid: 0.0% |
|  |  | IS: 0.3% to 0.6% |
| Recovery  (20, 200, and 800 pg/mL) | Pachymic acid | 82.8% to 97.6% |
|  | IS | 65.5% |
| Intra-day reproducibility  (10, 20, 200, and 800 pg/mL) | RE | LLOQ: -0.8% |
|  |  | Others: -15.0% to -3.9% |
|  | CV | LLOQ: 5.1% |
|  |  | Others: 1.8% to 4.9% |
| Inter-day reproducibility  (10, 20, 200, and 800 pg/mL) | RE | LLOQ: -9.3% to -0.8% |
|  |  | Others: -15.0% to 10.9% |
|  | CV | LLOQ: 5.1% to 9.5% |
|  |  | Others: 0.5% to 7.7% |
| Calibration curve  (10, 20, 50, 100, 200, 500, and 1000 pg/mL) | Correlation coefficient (*r*) | 0.9931 to 0.9964 |
|  | RE | LLOQ: -7.1% to -1.0% |
|  |  | Others: -13.9% to 15.0% |
| Stability in blood (20 and 800 pg/mL)  At room temperature for 2 h | RR | 103.1% and 91.8% |
| Stability in blood (20 and 800 pg/mL)  On ice for 2 h | RR | 103.1% and 93.4% |
| Short-term stability (20 and 800 pg/mL)  At room temperature (acceptable range: 1°C to 30°C) for 4 and25 h | RR | 4 h: 95.8% and 95.1% |
|  |  | 25 h: 85.9% and 93.8% |
| Post-preparative stability (20 and 800 pg/mL)  In an autosampler set at 4°C for 24 and 48 h | RR | 24 h: 92.4% and 88.4% |
|  |  | 48 h: 93.8% and 98.2% |
| Freeze-thaw stability (20 and 800 pg/mL)  In a deep freezer (acceptable range: −30°C to −10°C) after 3 cycles of freeze-thaw | RR | 108.8% and 114.0% |
| Long-term stability (20 and 800 pg/mL)  In a freezer (acceptable range: −30°C to −10°C) for 13, 39, and 228 days | RR | 13 days: 110.9% and 113.9% |
|  |  | 39 days: 104.1% and 107.6% |
|  |  | 228 days: 89.6% and 92.5% |
| Dilution integrity  (10-fold dilution) | RE | -2.9% |
|  | CV | 4.4% |
| Matrix effect | CV | Matrix factor: 14.4% |
|  |  | Matrix factor (IS): 10.7% |
| Carry-over | Ratio | Pachymic acid: 0.0% |
|  |  | IS: 0.0% |
| Stock solution stability (0.1 mg/mL)  In a refrigerator (acceptable range: 1°C to 8°C) for 54 and 129 days | RR | 54 days: 101.7% |
|  |  | 129 days: 110.1% |
| Working solution stability (0.1 and 10 ng/mL)  In a refrigerator (acceptable range: 1°C to 8°C) for 53 and 128 days | RR | 53 days: 109.1% and 101.1% |
|  |  | 128 days: 102.1% and 113.2% |

continued

1. Heptamethoxyflavone

| Validation items | Results | |
| --- | --- | --- |
| Selectivity | Ratio | Heptamethoxyflavone: 6.6% to 32.5% |
|  |  | IS: 0.3% to 0.6% |
| Recovery  (8, 80, and 320 pg/mL) | Heptamethoxyflavone | 65.7% to 75.5% |
|  | IS | 65.5% |
| Intra-day reproducibility  (4, 8, 80, and 320 pg/mL) | RE | LLOQ: 4.3% |
|  |  | Others: -3.4% to -0.5% |
|  | CV | LLOQ: 10.5% |
|  |  | Others: 2.6% to 12.9% |
| Inter-day reproducibility  (4, 8, 80, and 320 pg/mL) | RE | LLOQ: -7.8% to 4.3% |
|  |  | Others: -14.0% to 1.3% |
|  | CV | LLOQ: 10.4% to 13.9% |
|  |  | Others: 1.1% to 12.9% |
| Calibration curve  (4, 8, 20, 40, 80, 200, and 400 pg/mL) | Correlation coefficient (*r*) | 0.9937 to 0.9946 |
|  | RE | LLOQ: -2.3% to 4.0% |
|  |  | Others: -13.0% to 15.0% |
| Stability in blood (8 and 320 pg/mL)  At room temperature for 2 h | RR | 107.1% and 96.0% |
| Stability in blood (8 and 320 pg/mL)  On ice for 2 h | RR | 97.5% and 92.3% |
| Short-term stability (8 and 320 pg/mL)  At room temperature (acceptable range: 1°C to 30°C) for 4 and25 h | RR | 4 h: 99.5% and 103.3% |
|  |  | 25 h: 90.3% and 91.2% |
| Post-preparative stability (8 and 320 pg/mL)  In an autosampler set at 4°C for 24 and 48 h | RR | 24 h: 100.3% and 86.3% |
|  |  | 48 h: 96.5% and 90.8% |
| Freeze-thaw stability (8 and 320 pg/mL)  In a deep freezer (acceptable range: −30°C to −10°C) after 3 cycles of freeze-thaw | RR | 107.1% and 113.5% |
| Long-term stability (8 and 320 pg/mL)  In a freezer (acceptable range: −30°C to −10°C) for 13, 39, and 228 days | RR | 13 days: 90.3% and 113.1% |
|  |  | 39 days: 85.5% and 99.7% |
|  |  | 228 days: 85.0% and 87.5% |
| Dilution integrity  (10-fold dilution) | RE | -12.8% |
|  | CV | 1.9% |
| Matrix effect | CV | Matrix factor: 2.4% |
|  |  | Matrix factor (IS): 10.7% |
| Carry-over | Ratio | Heptamethoxyflavone: 10.7% |
|  |  | IS: 0.0% |
| Stock solution stability (0.1 mg/mL)  In a refrigerator (acceptable range: 1°C to 8°C) for 54 and 129 days | RR | 54 days: 98.7% |
|  |  | 129 days: 92.7% |
| Working solution stability (0.04 and 4 ng/mL)  In a refrigerator (acceptable range: 1°C to 8°C) for 53 and 128 days | RR | 53 days: 109.3% and 100.4% |
|  |  | 128 days: 101.6% and 98.8% |

continued

1. Naringenin

| Validation items | Results | |
| --- | --- | --- |
| Selectivity | Ratio | Naringenin: 0.5% to 8.5% |
|  |  | IS: 0.0% |
| Recovery  (100, 1000, and 4000 pg/mL) | Naringenin | 88.5% to 109.7% |
|  | IS | 23.4% |
| Intra-day reproducibility  (50, 100, 1000, and 4000 pg/mL) | RE | LLOQ: 15.4% |
|  |  | Others: 10.3% to 13.0% |
|  | CV | LLOQ: 8.0% |
|  |  | Others: 8.6% to 13.1% |
| Inter-day reproducibility  (50, 100, 1000, and 4000 pg/mL) | RE | LLOQ: 8.4% to 15.4% |
|  |  | Others: -14.3% to 13.0% |
|  | CV | LLOQ: 2.8% to 8.0% |
|  |  | Others: 3.2% to 13.1% |
| Calibration curve  (50, 100, 250, 500, 1000, 2500, and 5000 pg/mL) | Correlation coefficient (*r*) | 0.9964 to 0.9991 |
|  | RE | LLOQ: -6.0% to -2.6% |
|  |  | Others: -12.2% to 10.0% |
| Stability in blood (100 and 4000 pg/mL)  At room temperature for 2 h | RR | 99.8% and 93.2% |
| Stability in blood (100 and 4000 pg/mL)  On ice for 2 h | RR | 95.8% and 98.1% |
| Short-term stability (100 and 4000 pg/mL)  At room temperature (acceptable range: 1°C to 30°C) for 4 and25 h | RR | 4 h: 102.1% and 92.8% |
|  |  | 24 h: 113.8% and 112.2% |
| Post-preparative stability (100 and 4000 pg/mL)  In an autosampler set at 4°C for 24 and 48 h | RR | 25 h: 91.7% and 96.7% |
|  |  | 48 h: 85.6% and 87.1% |
| Freeze-thaw stability (100 and 4000 pg/mL)  In a deep freezer (acceptable range: −30°C to −10°C) after 3 cycles of freeze-thaw | RR | 90.6% and 96.7% |
| Long-term stability (100 and 4000 pg/mL)  In a freezer (acceptable range: −30°C to −10°C) for 22, 31, and 220 days | RR | 22 days: 101.9% and 95.8% |
|  |  | 31 days: 98.1% and 95.8% |
|  |  | 220 days: 86.8% and 85.2% |
| Dilution integrity  (10-fold dilution) | RE | -2.0% |
|  | CV | 7.1% |
| Matrix effect | CV | Matrix factor: 6.4% |
|  |  | Matrix factor (IS): 1.4% |
| Carry-over | Ratio | Naringenin: 6.1% |
|  |  | IS: 0.0% |
| Stock solution stability (0.5 mg/mL)  In a refrigerator (acceptable range: 1°C to 8°C) for 54 and 129 days | RR | 54 days: 97.5% |
|  |  | 129 days: 96.7% |
| Working solution stability (0.5 and 50 ng/mL)  In a refrigerator (acceptable range: 1°C to 8°C) for 53 and 128 days | RR | 53 days: 87.6% and 96.4% |
|  |  | 128 days: 102.6% and 99.8% |

continued

1. Nobiletin

| Validation items | Results | |
| --- | --- | --- |
| Selectivity | Ratio | Nobiletin: 5.8% to 26.6% |
|  |  | IS: 0.3% to 0.6% |
| Recovery  (8, 80, and 320 pg/mL) | Nobiletin | 62.7% to 70.1% |
|  | IS | 65.5% |
| Intra-day reproducibility  (4, 8, 80, and 320 pg/mL) | RE | LLOQ: -7.3% |
|  |  | Others: -9.9% to -3.1% |
|  | CV | LLOQ: 8.4% |
|  |  | Others: 1.3% to 8.9% |
| Inter-day reproducibility  (4, 8, 80, and 320 pg/mL) | RE | LLOQ: -9.5% to -5.0% |
|  |  | Others: -10.0% to -2.2% |
|  | CV | LLOQ: 6.5% to 9.0% |
|  |  | Others: 1.3% to 9.8% |
| Calibration curve  (4, 8, 20, 40, 80, 200, and 400 pg/mL) | Correlation coefficient (*r*) | 0.9910 to 0.9959 |
|  | RE | LLOQ: -5.8% to 3.5% |
|  |  | Others: -15.0% to 14.8% |
| Stability in blood (8 and 320 pg/mL)  At room temperature for 2 h | RR | 86.3% and 99.2% |
| Stability in blood (8 and 320 pg/mL)  On ice for 2 h | RR | 91.7% and 91.3% |
| Short-term stability (8 and 320 pg/mL)  At room temperature (acceptable range: 1°C to 30°C) for 4 and25 h | RR | 4 h: 106.5% and 109.7% |
|  |  | 25 h: 114.3% and 92.5% |
| Post-preparative stability (8 and 320 pg/mL)  In an autosampler set at 4°C for 24 and 48 h | RR | 24 h: 89.5% and 88.0% |
|  |  | 48 h: 87.7% and 89.7% |
| Freeze-thaw stability (8 and 320 pg/mL)  In a deep freezer (acceptable range: −30°C to −10°C) after 3 cycles of freeze-thaw | RR | 86.0% and 107.9% |
| Long-term stability (8 and 320 pg/mL)  In a freezer (acceptable range: −30°C to −10°C) for 13, 39, and 228 days | RR | 13 days: 91.2% and 114.0% |
|  |  | 39 days: 88.0% and 114.3% |
|  |  | 228 days: 89.9% and 103.2% |
| Dilution integrity  (10-fold dilution) | RE | -9.1% |
|  | CV | 3.1% |
| Matrix effect | CV | Matrix factor: 5.6% |
|  |  | Matrix factor (IS): 10.7% |
| Carry-over | Ratio | Nobiletin: 4.1% |
|  |  | IS: 0.0% |
| Stock solution stability (0.1 mg/mL)  In a refrigerator (acceptable range: 1°C to 8°C) for 54 and 129 days | RR | 54 days: 99.8% |
|  |  | 129 days: 89.1% |
| Working solution stability (0.04 and 4 ng/mL)  In a refrigerator (acceptable range: 1°C to 8°C) for 53 and 128 days | RR | 53 days: 95.0% and 102.6% |
|  |  | 128 days: 89.0% and 109.6% |

continued

1. Liquiritigenin

| Validation items | Results | |
| --- | --- | --- |
| Selectivity | Ratio | Liquiritigenin: 0.0% |
|  |  | IS: 0.0% |
| Recovery  (4, 40, 160 pg/mL) | Liquiritigenin | 85.6% to 92.5% |
|  | IS | 23.4% |
| Intra-day reproducibility  (2, 4, 40, and 160 pg/mL) | RE | LLOQ: 4.5% |
|  |  | Others: 1.9% to 6.5% |
|  | CV | LLOQ: 8.1% |
|  |  | Others: 3.6% to 5.0% |
| Inter-day reproducibility  (2, 4, 40, and 160 pg/mL) | RE | LLOQ: 0.5% to 6.0% |
|  |  | Others: -8.8% to 7.0% |
|  | CV | LLOQ: 8.1% to 14.9% |
|  |  | Others: 3.2% to 14.7% |
| Calibration curve  (2, 4, 10, 20, 40, 100, and 200 pg/mL) | Correlation coefficient (*r*) | 0.9990 to 0.9996 |
|  | RE | LLOQ: -4.0% to -1.5% |
|  |  | Others: -4.0% to 8.0% |
| Stability in blood (4 and 160 pg/mL)  At room temperature for 2 h | RR | 82.0% and 101.4% |
| Stability in blood (4 and 160 pg/mL)  On ice for 2 h | RR | 91.6% and 99.5% |
| Short-term stability (4 and 160 pg/mL)  At room temperature (acceptable range: 1°C to 30°C) for 4 and25 h | RR | 4 h: 101.0% and 93.1% |
|  |  | 24 h: 108.6% and 100.0% |
| Post-preparative stability (4 and 160 pg/mL)  In an autosampler set at 4°C for 24 and 48 h | RR | 25 h: 89.2% and 99.3% |
|  |  | 48 h: 94.1% and 103.4% |
| Freeze-thaw stability (4 and 160 pg/mL)  In a deep freezer (acceptable range: −30°C to −10°C) after 3 cycles of freeze-thaw | RR | 105.0% and 92.5% |
| Long-term stability (4 and 160 pg/mL)  In a freezer (acceptable range: −30°C to −10°C) for 22, 31, and 220 days | RR | 22 days: 106.0% and 92.5% |
|  |  | 31 days: 94.2% and 87.4% |
|  |  | 220 days: 110.8% and 87.9% |
| Dilution integrity  (10-fold dilution) | RE | -13.1% |
|  | CV | 8.8% |
| Matrix effect | CV | Matrix factor: 3.3% |
|  |  | Matrix factor (IS): 1.4% |
| Carry-over | Ratio | Liquiritigenin: 3.7% |
|  |  | IS: 0.0% |
| Stock solution stability (0.1 mg/mL)  In a refrigerator (acceptable range: 1°C to 8°C) for 54 and 129 days | RR | 54 days: 99.6% |
|  |  | 129 days: 103.5% |
| Working solution stability (0.02 and 2 ng/mL)  In a refrigerator (acceptable range: 1°C to 8°C) for 53 and 128 days | RR | 53 days: 96.4% and 100.0% |
|  |  | 128 days: 108.0% and 110.9% |

continued

1. Isoliquiritigenin

| Validation items | Results | |
| --- | --- | --- |
| Selectivity | Ratio | Isoliquiritigenin: 0.0% to 8.5% |
|  |  | IS: 0.0% |
| Recovery  (4, 40, 160 pg/mL) | Isoliquiritigenin | 81.7% to 84.3% |
|  | IS | 23.4% |
| Intra-day reproducibility  (2, 4, 40, and 160 pg/mL) | RE | LLOQ: 12.0% |
|  |  | Others: 9.5% to 14.5% |
|  | CV | LLOQ: 12.2% |
|  |  | Others: 11.8% to 14.9% |
| Inter-day reproducibility  (2, 4, 40, and 160 pg/mL) | RE | LLOQ: 5.0% to 12.0% |
|  |  | Others: -6.9% to 14.5% |
|  | CV | LLOQ: 12.2% to 14.8% |
|  |  | Others: 4.1% to 14.9% |
| Calibration curve  (2, 4, 10, 20, 40, 100, and 200 pg/mL) | Correlation coefficient (*r*) | 0.9938 to 0.9993 |
|  | RE | LLOQ: -6.0% to -1.0% |
|  |  | Others: -11.5% to 14.0% |
| Stability in blood (4 and 160 pg/mL)  At room temperature for 2 h | RR | 81.0% and 46.8% |
| Stability in blood (4 and 160 pg/mL)  On ice for 2 h | RR | 103.0% and 90.2% |
| Short-term stability (4 and 160 pg/mL)  At room temperature (acceptable range: 1°C to 30°C) for 4 and25 h | RR | 4 h: 97.0% and 85.2% |
|  |  | 24 h: 96.5% and 101.2% |
| Post-preparative stability (4 and 160 pg/mL)  In an autosampler set at 4°C for 24 and 48 h | RR | 25 h: 110.0% and 115.2% |
|  |  | 48 h: 88.0% and 93.8% |
| Freeze-thaw stability (4 and 160 pg/mL)  In a deep freezer (acceptable range: −30°C to −10°C) after 3 cycles of freeze-thaw | RR | 95.3% and 88.3% |
| Long-term stability (4 and 160 pg/mL)  In a freezer (acceptable range: −30°C to −10°C) for 22, 31, and 220 days | RR | 22 days: 99.0% and 95.0% |
|  |  | 31 days: 105.0% and 87.7% |
|  |  | 220 days: 102.8% and 85.5% |
| Dilution integrity  (10-fold dilution) | RE | -6.9% |
|  | CV | 14.6% |
| Matrix effect | CV | Matrix factor: 4.1% |
|  |  | Matrix factor (IS): 1.4% |
| Carry-over | Ratio | Isoliquiritigenin: 11.9% |
|  |  | IS: 0.0% |
| Stock solution stability (0.1 mg/mL)  In a refrigerator (acceptable range: 1°C to 8°C) for 54 and 129 days | RR | 54 days: 96.9% |
|  |  | 129 days: 96.9% |
| Working solution stability (0.02 and 2 ng/mL)  In a refrigerator (acceptable range: 1°C to 8°C) for 53 and 128 days | RR | 53 days: 89.9% and 92.7% |
|  |  | 128 days: 108.2% and 102.2% |

continued

1. 18β-Glycyrrhetinic acid

| Validation items | Results | |
| --- | --- | --- |
| Selectivity | Ratio | Glycyrrhetinic acid: 1.9% to 26.6% |
|  |  | IS: 0.3% to 0.6% |
| Recovery  (1600, 16000, and 64000 pg/mL) | Glycyrrhetinic acid | 84.0% to 93.6% |
|  | IS | 65.5% |
| Intra-day reproducibility  (800, 1600, 16000, and 64000 pg/mL) | RE | LLOQ: -11.3% |
|  |  | Others: -12.5% to -10.0% |
|  | CV | LLOQ: 7.3% |
|  |  | Others: 1.3% to 3.1% |
| Inter-day reproducibility  (800, 1600, 16000, and 64000 pg/mL) | RE | LLOQ: -11.3% to -6.6% |
|  |  | Others: -14.1% to 8.3% |
|  | CV | LLOQ: 3.4% to 7.3% |
|  |  | Others: 0.8% to 6.8% |
| Calibration curve  (800, 1600, 4000, 8000, 16000, 40000, and 80000 pg/mL) | Correlation coefficient (*r*) | 0.9930 to 0.9974 |
|  | RE | LLOQ: -3.5% to 5.4% |
|  |  | Others: -14.4% to 14.9% |
| Stability in blood (1600 and 64000 pg/mL)  At room temperature for 2 h | RR | 104.2% and 98.2% |
| Stability in blood (1600 and 64000 pg/mL)  On ice for 2 h | RR | 108.8% and 99.1% |
| Short-term stability (1600 and 64000 pg/mL)  At room temperature (acceptable range: 1°C to 30°C) for 4 and25 h | RR | 4 h: 89.0% and 98.9% |
|  |  | 25 h: 90.2% and 105.0% |
| Post-preparative stability (1600 and 64000 pg/mL)  In an autosampler set at 4°C for 24 and 48 h | RR | 24 h: 106.0% and 104.9% |
|  |  | 48 h: 110.8% and 111.4% |
| Freeze-thaw stability (1600 and 64000 pg/mL)  In a deep freezer (acceptable range: −30°C to −10°C) after 3 cycles of freeze-thaw | RR | 92.3% and 112.0% |
| Long-term stability (1600 and 64000 pg/mL)  In a freezer (acceptable range: −30°C to −10°C) for 13, 39, and 228 days | RR | 13 days: 102.6% and 113.1% |
|  |  | 39 days: 108.3% and 104.4% |
|  |  | 228 days: 113.5% and 109.2% |
| Dilution integrity  (10-fold dilution) | RE | -4.8% |
|  | CV | 0.9% |
| Matrix effect | CV | Matrix factor: 2.5% |
|  |  | Matrix factor (IS): 10.7% |
| Carry-over | Ratio | Glycyrrhetinic acid: 18.6% |
|  |  | IS: 0.0% |
| Stock solution stability (0.8 mg/mL)  In a refrigerator (acceptable range: 1°C to 8°C) for 54 and 129 days | RR | 54 days: 100.0% |
|  |  | 129 days: 100.0% |
| Working solution stability (8 and 800 ng/mL)  In a refrigerator (acceptable range: 1°C to 8°C) for 53 and 128 days | RR | 53 days: 108.4% and 100.7% |
|  |  | 128 days: 96.9% to 103.4% |
