# Supplementary material for: Pharmacokinetic Profiles of Active Ingredients and Its Metabolites Derived from Rikkunshito, a Ghrelin Enhancer, in Healthy Japanese Volunteers: A Cross-Over, Randomized Study
Source: PLoS One. 2015 Jul 17;10(7):e0133159. doi: 10.1371/journal.pone.0133159 (PMC4506051; doi:10.1371/journal.pone.0133159)
Supplement: S9 Table — (DOCX) [file pone.0133159.s013.docx]

**S8 Table. Dose proportionality test using power model.**

| Compound | Parameter | β | 90% Lower CI | 90% Upper CI | Inference |
| --- | --- | --- | --- | --- | --- |
| Atractylodin | *C*_max_ | 0.988 | 0.802 | 1.17 | Linear^a^ |
|  | AUC*_0-last_* | 1.60 | 1.38 | 1.82 | Nonlinear |
| Atractylodin carboxylic acid | *C*_max_ | 1.05 | 0.918 | 1.19 | Linear |
|  | AUC*_0-last_* | 1.08 | 0.990 | 1.17 | Linear |
| Pachymic acid | *C*_max_ | 0.856 | 0.687 | 1.03 | Nonlinear |
|  | AUC*_0-last_* | 1.39 | 1.15 | 1.64 | Nonlinear |
| Heptamethoxyflavone | *C*_max_ | 0.817 | 0.628 | 1.01 | Nonlinear |
|  | AUC*_0-last_* | 0.804 | 0.486 | 1.12 | Nonlinear |
| Naringenin | *C*_max_ | 1.06 | 0.735 | 1.38 | Nonlinear |
|  | AUC*_0-last_* | 1.02 | 0.663 | 1.37 | Nonlinear |
| Nobiletin | *C*_max_ | 0.701 | 0.395 | 1.01 | Nonlinear |
|  | AUC*_0-last_* | 0.604 | -0.0903 | 1.30 | Nonlinear |
| Liquiritigenin | *C*_max_ | 1.11 | 0.911 | 1.30 | Nonlinear |
|  | AUC*_0-last_* | 1.12 | 0.981 | 1.26 | Nonlinear |
| Isoliquiritigenin | *C*_max_ | 0.692 | 0.463 | 0.921 | Nonlinear |
|  | AUC*_0-last_* | 1.27 | 0.956 | 1.59 | Nonlinear |
| 18β-Glycyrrhetinic acid | *C*_max_ | 0.819 | 0.652 | 0.987 | Nonlinear |
|  | AUC*_0-last_* | 0.979 | 0.847 | 1.11 | Linear |

CI; confidence interval.

^a^ The system was considered to be linear when the 90% CI of β was included 1, CI_lower_ ≥0.8, and CI_upper_ ≤1.25.
