# Supplementary material for: Pharmacokinetic Profiles of Active Ingredients and Its Metabolites Derived from Rikkunshito, a Ghrelin Enhancer, in Healthy Japanese Volunteers: A Cross-Over, Randomized Study
Source: PLoS One. 2015 Jul 17;10(7):e0133159. doi: 10.1371/journal.pone.0133159 (PMC4506051; doi:10.1371/journal.pone.0133159)
Supplement: S10 Table — (DOCX) [file pone.0133159.s014.docx]

**S9 Table. Stability of 32 ingredients in gastric pH solution.**

| Compound | Quantitative value (ng/mL) | | | |
| --- | --- | --- | --- | --- |
|  | 0.25 h | 0.5 h | 1 h | 2 h |
| [6]-Gingerol | 154 | 152 | 154 | 149 |
| [8]-Gingerol | 13.5 | 13.1 | 12.7 | 12.7 |
| [10]-Gingerol | 7.00 | 8.07 | 7.53 | 7.62 |
| [6]-Shogaol | 18.1 | 17.9 | 18.5 | 17.9 |
| [8]-Shogaol | 2.16 | 2.00 | 1.89 | 1.83 |
| Ginsenoside Rb_1_ | 371 | 297 | 202 | 86.4 |
| Ginsenoside Rb_2_ | 313 | 275 | 168 | 77.3 |
| Ginsenoside Rc | 219 | 188 | 115 | 39.3 |
| Ginsenoside Rd | 122 | 98.5 | 68.1 | 20.3 |
| Ginsenoside Re | 123 | 96.7 | 58.4 | 23.7 |
| Ginsenoside Rf | 172 | 190 | 159 | 113 |
| Ginsenoside Rg_1_ | 234 | 174 | 118 | 41.6 |
| Ginsenoside Rg_2_ | 107 | 121 | 157 | 126 |
| Pachymic acid | 42.8 | 40.2 | 46.5 | 28.3 |
| Liquiritin apioside | 1440 | 1250 | 1340 | 1190 |
| Liquiritin | 1750 | 1670 | 1830 | 1720 |
| Isoliquiritigenin | 16.0 | 15.2 | 15.5 | 15.2 |
| Glycycoumarin | 5.85 | 5.58 | 7.09 | 5.09 |
| 18β-Glycyrrhetinic acid | 0.443 | 0.371 | 0.485 | 0.587 |
| Glycyrrhetinic acid 3-*O*-glucuronide | 1.75 | 1.56 | 1.80 | 1.67 |
| Atractylodin | 9.64 | 9.03 | 9.72 | 8.13 |
| Hesperidin | 3250 | 2860 | 3130 | 2570 |
| Hesperetin | 4.42 | 4.38 | 4.36 | 3.90 |
| Nobiletin | 17.8 | 18.1 | 21.5 | 19.6 |
| Narirutin | 1870 | 1630 | 1700 | 1690 |
| Tangeretin | 5.66 | 5.50 | 6.62 | 5.95 |
| Heptamethoxyflavone | 25.6 | 25.5 | 30.7 | 28.7 |
| Synephrine | 3140 | 3200 | 3180 | 2960 |
| Naringin | 1120 | 959 | 1060 | 986 |
| Naringenin | 2120 | 2030 | 2140 | 1990 |
| PTH-15 | 78.1 | 76.4 | 43.7 | 46.5 |
| Oleanolic acid | 5.06 | 3.61 | 3.54 | 3.28 |

Rikkunshito gradually dissolved in acidic solution (pH 1.2) at 37°C, and the concentration of atractylodin after adding 0.25, 0.5, 1, and 2 h was measured by gas chromatography–mass spectrometry.
