# Supplementary material for: Pharmacokinetic Profiles of Active Ingredients and Its Metabolites Derived from Rikkunshito, a Ghrelin Enhancer, in Healthy Japanese Volunteers: A Cross-Over, Randomized Study
Source: PLoS One. 2015 Jul 17;10(7):e0133159. doi: 10.1371/journal.pone.0133159 (PMC4506051; doi:10.1371/journal.pone.0133159)
Supplement: S11 Table — (DOCX) [file pone.0133159.s015.docx]

**S11 Table. Formation of ingredients derived from rikkunshito in formulation, blood, and urine.**

| Formulation | Metabolite by enterobacterium | Blood | Urine |
| --- | --- | --- | --- |
| Atractylodin | N/A | Atractylodin  Atractylodin carboxylic acid^a^ | Atractylodin |
| Pachymic acid | N/A | Pachymic acid | BQL |
| Heptamethoxyflavone | N/A | Heptamethoxyflavone | BQL |
| Narirutin | Naringenin | Naringenin  Conjugate metabolites of naringenin^a^ | Naringenin |
| Naringin | Naringenin (portion) | Naringin  Naringenin  Conjugate metabolites of naringenin^a^ | Naringin  Naringenin |
| Naringenin | N/A | Naringenin  Conjugate metabolites of naringenin^a^ | Naringenin |
| Nobiletin | N/A | Nobiletin | BQL |
| Liquiritin | Liquiritigenin^b^ | Liquiritin  Liquiritigenin^c^ | Liquiritin  Liquiritigenin^c^ |
| Liquiritin apioside | Liquiritigenin^b^ | Liquiritin apioside Liquiritigenin^c^ | Liquiritin apioside Liquiritigenin^c^ |
| Liquiritigenin | N/A | Liquiritigenin^c^ | Liquiritigenin^c^ |
| Isoliquiritin | Isoliquiritigenin | Isoliquiritigenin Conjugate metabolites of isoliquiritigenin | Isoliquiritigenin Conjugate metabolites of isoliquiritigenin |
| Isoliquiritin apioside | Isoliquiritigenin | Isoliquiritigenin Conjugate metabolites of isoliquiritigenin | Isoliquiritigenin Conjugate metabolites of isoliquiritigenin |
| Isoliquiritigenin | N/A | Isoliquiritigenin Conjugate metabolites of isoliquiritigenin | Isoliquiritigenin Conjugate metabolites of isoliquiritigenin |
| Glycyrrhizic acid | 18β-Glycyrrhetinic acid | 18β-Glycyrrhetinic acid  Glycyrrhetinic acid 3-*O*-glucuronide | 18β-Glycyrrhetinic acid  Glycyrrhetinic acid 3-*O*-glucuronide |
| 18β-Glycyrrhetinic acid | N/A | 18β-Glycyrrhetinic acid  Glycyrrhetinic acid 3-*O*-glucuronide | 18β-Glycyrrhetinic acid  Glycyrrhetinic acid 3-*O*-glucuronide |
| Hesperidin | Hesperetin | Conjugate metabolites of hesperetin | Hesperidin  Hesperetin  Conjugate metabolites of hesperetin |

BQL; below the quantification limit.

^a^It was not analyzed in urine samples.

^b^It was cited from previous reports [Li C, Homma M, Oka K. Characteristics of delayed excretion of flavonoids in human urine after administration of Shosaiko-to, a herbal medicine. Biol Pharm Bull. 1998; 21: 1251-7.].

^c^Although in this study was not measured, according to the above reports, it will also enter the blood and urine in this study.
